# Host-driven remodeling of rumen microbiota supports lactation metabolism in buffalo

**DOI:** 10.3389/fmicb.2025.1617388

**Published:** 2025-06-25

**Authors:** Zixu Ding, Yixue Xu, Yan Wang, Miaoer Liu, Peng Zhu, Kuiqing Cui, Chunyan Yang, Changlong Xu, Tong Feng, Qingyou Liu

**Affiliations:** ^1^Guangdong Provincial Key Laboratory of Animal Molecular Design and Precise Breeding, School of Life Science and Engineering, Foshan University, Foshan, China; ^2^Guangdong Provincial Key Laboratory of Animal Nutrition Control, College of Animal Science, South China Agricultural University, Guangzhou, China; ^3^State Key Laboratory for Conservation and Utilization of Subtropical Agro-Bioresources, College of Animal Science and Technology, Guangxi University, Nanning, China; ^4^Guangxi Key Laboratory of Beibu Gulf Marine Biodiversity Conservation, Beibu Gulf Marine Ecological Environment Field Observation and Research Station of Guangxi, Beibu Gulf University, Qinzhou, China; ^5^Guangxi Zhuang Autonomous Region Buffalo Research Institute, Nanning, China; ^6^The Reproductive Medical Center of Nanning Second People’s Hospital, Nanning, China; ^7^Key Laboratory of Molecular Biophysics of the Ministry of Education, Hubei Key Laboratory of Bioinformatics and Molecular Imaging, Center for Artificial Biology, College of Life Science and Technology, Department of Bioinformatics and Systems Biology, Huazhong University of Science and Technology, Wuhan, China

**Keywords:** buffalo, multiomics, metagenomic, metabolomic, lactation

## Abstract

**Introduction:**

Rumen microbiota and host metabolites play a key role in regulating ruminant production performance and physiological adaptation. However, the interplay between host physiological status and rumen microbial-metabolite dynamics across lactation stages in buffaloes remains unclear.

**Methods:**

This study employed a multi-omics approach, integrating metagenomic and serum metabolomic analyses, to investigate microbial remodeling and metabolic adaptations in buffaloes during lactation and dry periods.

**Results:**

Metagenomic analysis revealed increased abundances of *Anaerovibrio*, *Succiniclasticum*, and *Methanobrevibacter_A* during lactation, associated with lipid hydrolysis, propionate production, and methanogenesis, respectively. Glycoside hydrolase families GH2, GH3, GH5, and GH13 were enriched, indicating elevated carbohydrate degradation potential. In contrast, *Butyrivibrio*, *Fibrobacter*, and *Eubacterium_Q* were predominant during the dry period, contributing to fiber degradation and butyrate synthesis. Functional pathways related to niacin metabolism, bicarbonate reabsorption, and neuroactive ligand-receptor interaction were significantly upregulated during lactation. Metabolomic profiling identified lactation-enriched metabolites such as indole-3-methylacetate, D-maltose, and gluconic acid, correlating with immune and metabolic indicators (e.g., IgA, glucose, LDL). Conversely, dry period metabolites such as 1-methylhistidine and 5-hydroxyindoleacetic acid indicated physiological shifts toward tissue repair and stress mitigation.

**Discussion:**

The integrative analysis revealed that host physiological demands during lactation coordinate rumen microbial restructuring to enhance triglyceride degradation, fatty acid biosynthesis, and energy mobilization, thereby supporting milk production. These findings provide novel insights into the host-driven microbiome-metabolite axis underlying lactation in buffaloes.

## Introduction

Buffaloes (*Bubalus bubalis*) are an important livestock species valued for their contributions to milk and meat production. In certain regions, particularly across Asia and parts of Africa, they also continue to serve as sources of agricultural draft power within traditional farming systems (Zhang et al., 2020). With a global population exceeding 200 million, buffaloes support the livelihoods of more than two billion people (Di Stasio and Brugiapaglia, 2021) and contribute over 15% to the world’s annual milk production (Piotin et al., 2023). Buffalo milk is a highly nutritious food, with both its protein content and lactose percentage significantly higher than those of cow milk and goat milk (La Torre et al., 2024). Furthermore, buffalo milk contains markedly higher total fatty acids (8467.77 ± 746.18 mg/100 g) compared to cow milk (925.27 ± 173.47 mg/100 g) (Li et al., 2020). Fatty acids play critical roles in various physiological functions, including body weight regulation and the maintenance of gut health (Hernández et al., 2019). Recent studies suggest that the unique lipid and protein profiles of buffalo milk may contribute to improved metabolic health and immune function in humans (Vargas-Ramella et al., 2021). Consequently, research on buffalo milk is highly relevant to human dietary health and nutrition.

The rumen microbiome plays a central role in ruminant digestion and nutrient metabolism. Although feeding conditions and host genetics are well-established factors influencing rumen microbial composition, increasing evidence highlights the critical influence of the host’s physiological state. For instance, previous studies have shown that under comparable dietary regimes, differences in alpha diversity among cattle breeds such as Angus, Charolais, and Kinsella hybrids are minimal (Li et al., 2019). Furthermore, while variations in dietary protein levels can shift the abundance of certain microbial taxa, they do not significantly alter core rumen microbial functions (Belanche et al., 2012). In contrast, physiological stress or disease-induced changes in host metabolic demand can lead to substantial restructuring of the rumen microbiome, characterized by the enrichment or depletion of specific functional groups (Round and Mazmanian, 2009). While diet remains the dominant factor shaping rumen microbiota, recent studies suggest that host physiological changes, including the transition from lactation to the dry period, also play a crucial role in modulating microbial composition and function (Tröscher-Mußotter et al., 2022). These physiological changes entail altered metabolic demands, potentially influencing not only microbial activity but also host-microbe metabolic interactions. Therefore, to obtain a comprehensive understanding of rumen function, it is essential to consider both dietary inputs and host physiological context. In this study, metagenomic techniques enabled in-depth taxonomic and functional profiling, while metabolomic analyses provided complementary insights into the biochemical outcomes of host–microbiota interactions.

The lactation and dry period are two critical stages in the milk production cycle of ruminants (Cook, 2019; Shortall et al., 2018). Previous studies have used high-throughput sequencing to analyze microbial diversity and functional roles in the ruminant gastrointestinal tract during these phases (Sun et al., 2020). In dairy cattle, species such as *Prevotella* and *Bacteroides* are more abundant in the rumen during lactation than in the dry period (Xue et al., 2020). Similarly, studies on goats have shown increased abundances of *Prevotellaceae* and *Lachnospiraceae* during lactation (de Sant’ana et al., 2022). Based on these findings, we speculate whether the host drives microbial remodeling to adapt to lactation needs.

Ruminal microorganisms play a pivotal role in nutrient metabolism and production performance in ruminants. They are essential for the degradation of cellulose, the generation of volatile fatty acids (VFAs) that serve as major energy sources, and the synthesis of amino acids and other bioactive compounds (Adeyemi et al., 2015; Hua et al., 2017; Ozbayram et al., 2020; Wang et al., 2016). While the rumen microbiota of dairy cattle and goats has been extensively studied, the functional dynamics of the buffalo rumen microbiome across different physiological stages—particularly during lactation and the dry period—remain insufficiently explored (Wallace et al., 2019). Moreover, the broader impact of rumen microorganisms on host systemic metabolism is still not fully understood. In this context, serum metabolomic profiling provides a valuable window into host metabolic alterations, offering an important tool for elucidating the interplay between microbial activity and host physiology (Zhao et al., 2021). During lactation, cows experience a heightened demand for glucose, amino acids, and lipids to support milk synthesis, which is often accompanied by mobilization of body reserves and shifts in endocrine regulation. These changes are mirrored in the serum metabolome, with elevated levels of metabolites associated with energy metabolism, such as β-hydroxybutyrate, non-esterified fatty acids (NEFAs), and certain amino acids (Ghaffari et al., 2024). Despite these insights, no studies to date have systematically integrated ruminal metagenomic profiles with serum metabolomic data in buffaloes across these distinct physiological stages. Therefore, exploring the relationship between the ruminal microbiome and host serum metabolites across lactation and the dry period is essential for improving our understanding of host–microbiota metabolic interactions.

In the present study, we applied an integrated multi-omics approach combining rumen metagenomics and serum metabolomics to characterize changes in microbial composition, function, and host metabolic profiles across the lactation and dry periods in buffaloes. These findings provide molecular insights into the metabolic adaptations underlying buffalo lactation and offer valuable information for the advancement of buffalo nutrition and dairy production strategies.

## Methods

### Animal management

A total of 70 buffaloes were selected from the Guangxi Buffalo Research Institute farm, including: Lactating group (*n* = 35): Mid-lactation (90–150 days in milk, DIM), aged 4–6 years, 2–3 parity, body weight (BW) 550–650 kg, average milk yield 7.86 ± 1.3 kg/day. Dry group (*n* = 35): ≥60 days post-drying, aged 5–7 years, 3–4 parity, BW 500–600 kg, with last lactation yield recorded as 2,133 ± 330 kg/year. All animals had a body condition score (BCS) between 3.0 and 3.5 (on a 5-point scale) and were confirmed healthy via veterinary examination (no signs of mastitis, lameness, or metabolic disorders). Buffaloes were housed in a naturally ventilated barn, each animal had an individual feeding space of 3.0 × 4.5 m and had free access to clean water. The barn maintained a temperature range of 18–28°C and a relative humidity of 60–75%, both of which were monitored daily to ensure stable environmental conditions.

Milking was performed twice daily at 05:00 and 16:00 using portable bucket milking units (Model MB-300, Italy), with a pulsation rate of 60 cycles/min and vacuum pressure maintained at 42 kPa, in compliance with ISO 6690:2007 standards for buffalo milking. All buffaloes received ad libitum access to a total mixed ration (TMR) formulated according to NRC (2001) nutrient requirements (Yu et al., 2003). The lactating group was offered 28 kg DM/head/day, whereas the dry group received 20 kg DM/head/day, with feed residues maintained at 10–15% of offered feed. Water intake was monitored using stainless steel automatic drinkers (AquaFlow BF-200 series) with digital flow meters (±2% accuracy), yielding mean ± SD daily values of 62.4 ± 5.3 L for lactating and 41.7 ± 4.2 L for dry buffaloes. All feed ingredients were sourced and prepared by the Guangxi Buffalo Research Institute. The diet of buffaloes during the lactation and dry lactation periods (Table 1). Nutrition composition of the diet (Table 2).

**Table 1:** Diet formula table for lactation period buffaloes and dry period buffaloes.

Lactation period	
Name	Content (%)
*Pennisetum purpureum*	35.70
Peanut vine	9.50
Marigold	9.50
Jasmine residue	11.90
Distiller’s grains	28.70
Pal kernel expeller	4.30
Premix	0.20
Salt	0.20

Dry period	
Name	Content (%)
*Pennisetum purpureum*	50.10
Peanut vine	4.80
Marigold	8.00
Jasmine residue	10.00
Distiller’s grains	23.10
Pal kernel expeller	3.60
Premix	0.20
Salt	0.20

**Table 2:** Nutrition composition of the diet for lactation period buffaloes and dry period buffaloes.

Lactation period	
Name	Content (%)
Dry matter (DM)	90.00
Crude protein (CP)	14.20
Neutral detergent fiber (NDF)	45.50
Acid detergent fiber (ADF)	28.70
Ether extract (EE)	3.80
Ash	6.50
Calcium (Ca)	0.65
Phosphorus (P)	0.40
Metabolizable energy (ME)	10.20

Dry period	
Name	Content (%)
Dry matter (DM)	88.50
Crude protein (CP)	11.80
Neutral detergent fiber (NDF)	52.30
Acid detergent fiber (ADF)	34.10
Ether extract (EE)	3.20
Ash	6.10
Calcium (Ca)	0.60
Phosphorus (P)	0.35
Metabolizable energy (ME)	8.70

While the dietary formulations differed between lactating and dry periods, these variations were intentionally designed to meet the distinct nutritional requirements of each physiological stage according to NRC (2001) guidelines. NDF/CP and Ca/P were maintained within consistent ranges to minimize dietary variation between the two groups (Gebrehawariat et al., 2010).

### Metagenomic sample collection and serum sample collection

A total of 62 samples were subjected to metagenomic sequencing (Supplementary Table S1), including 29 samples from lactating buffaloes and 33 from dry buffaloes. Although 70 animals were initially enrolled, eight samples were excluded due to either poor DNA quality or insufficient yield, as determined by agarose gel electrophoresis and spectrophotometric analysis, or because rumen fluid collection failed due to animal noncompliance during sampling. Prior to collection, all buffaloes were fasted for 12 h. Rumen samples were then obtained via a stomach tube, immediately frozen in liquid nitrogen, and stored at −80°C until DNA extraction. For the serum samples, nine lactation period buffaloes and nine dry period buffaloes were randomly selected (Supplementary Table S2). Blood was drawn from the tail vein, placed in 5 mL blood collection tubes, and centrifuged at 4°C and 3,000 × g for 15 min. The serum was separated: 1 mL was used for biochemical index testing, and the remaining serum was stored at −80°C in 1.5 mL centrifuge tubes for subsequent metabolomics analysis.

### DNA extraction, metagenomic sequencing and metagenomics data processing

Genomic DNA was extracted from 3 g of rumen content using a modified phenol–chloroform method optimized for metagenomic applications. Samples were subjected to bead-beating (Mini-BeadBeater-24, Biospec Products, United States) using a 3:1 mixture of 0.1 mm and 0.5 mm zirconia beads in CTAB buffer, followed by phenol-chloroform extraction and ethanol precipitation. The resulting DNA pellets were resuspended in 50 μL of Tris-EDTA buffer. DNA purity (A260/A280>1.8) was assessed with a NanoPhotometer^®^ (IMPLEN, United States), and concentrations were further validated using a Qubit^®^ 2.0 fluorometer (Life Technologies, United States). All DNA samples were stored at −80°C until further processing. This classical DNA extraction method was preferred over commercial kits owing to its demonstrated effectiveness in recovering high-molecular-weight DNA and minimizing taxonomic bias in complex rumen microbial ecosystems, particularly those involving low-abundance and recalcitrant taxa.

Libraries were prepared using Illumina TruSeq DNA PCR-Free Kit (500 ng input DNA, Illumina, 15026486 Rev. C) with size selection for ~550 bp inserts, and sequenced on NovaSeq 6000 (2 × 150 bp, Q30 >85%), employing a read length of 150 base pairs (PE150). Raw reads were processed through: (1) Trimmomatic v0.35 (ILLUMINACLIP: TruSeq3-PE. fa:2:30:10, SLIDINGWINDOW:4:20, MINLEN:110) (Bolger et al., 2014); (2) Host DNA removal via Bowtie2 v2.3.3 (--very-sensitive-local) against *Bubalus bubalis* genome (ARS_UBD_2.0) and feed plant genomes; (3) Metagenome assembly using MegaHit v1.2.9 (--kmin-1pass --k-list 21, 33, 55, 77, 99, 127) with contig filtering (>1 kb, >5 × coverage). Metagenome-assembled genomes (MAGs) were generated through MetaBAT2/MaxBin2/CONCOCT binning and refined using DAS Tool (CheckM quality thresholds: >50% completeness, <10% contamination) (Langmead and Salzberg, 2012).

### Taxonomic and functional annotation from rumen microbial metagenomes

In this study, we employed our previously published water buffalo intestinal microbial genome catalog (comprising 4,960 strain-level metagenome-assembled genomes, MAGs) from Nature Communications as the reference genome database for relative abundance quantification and functional profiling (Tong et al., 2022). This catalog was constructed using 695 intestinal metagenomic samples with comprehensive metadata, ensuring high host-specific compatibility and precise strain-level resolution for water buffalo.

For taxonomic analysis, we performed strain-level relative abundance estimation using Salmon against the custom water buffalo MAG reference. Higher taxonomic ranks (genus to phylum) were aggregated based on Genome Taxonomy Database (GTDB) annotations (Lesker et al., 2020). The resulting abundance matrix was subjected to differential analysis via LEfSe (Linear Discriminant Analysis Effect Size), employing the Wilcoxon rank-sum test (*α* = 0.05) to identify taxa with significant abundance variations across groups (Cao et al., 2023). Functional annotation of MAGs followed a standardized metagenomic workflow:

Gene prediction: Protein-coding sequences were identified using Prokka with default parameters (Jin et al., 2023).
Functional annotation: Predicted proteins were classified into Clusters of Orthologous Groups (COGs) and Kyoto Encyclopedia of Genes and Genomes (KEGG) pathways using the eggNOG-mapper (v5.0) against the eggNOG database (Huerta-Cepas et al., 2016).
Enzyme annotation: Carbohydrate-active enzymes (CAZymes) were annotated through homology searches against the CAZy database using dbCAN2 with a defined E-value threshold (Zhang et al., 2018).

### Analysis of serum biochemical indices

Serum samples (200 μL) were analyzed using a URIT8030 discrete automatic biochemical analyzer (Xiamen Hafei Biotechnology) with commercial kits (Guilin Unite Medical Electronics) to measure 20 biochemical indices: high-density lipoprotein (HDL), low-density lipoprotein (LDL), glucose (GLU), immunoglobulin A (IgA), total cholesterol (CHOL), urea, triglycerides (TG), aspartate aminotransferase (AST), alanine aminotransferase (ALT), creatinine (Crea), uric acid (UA), total protein (TP), total bilirubin (TBIL), albumin (ALB), globulin (GLB), calcium (Ca), iron (Fe), magnesium (Mg), and phosphorus (P). All assays were performed following manufacturer’s protocols with internal quality controls, including daily calibration using provided standards and duplicate measurements for 10% randomly selected samples to ensure data reliability.

### Metabolomic sample preparation and serum metabolomic analysis

The analysis was conducted according to the method outlined by Sun et al. (2022). Serum samples were carefully thawed at 4°C overnight to prevent metabolite degradation, and for each sample, a 100 μL aliquot was precisely measured using calibrated pipettes and mixed with 300 μL of ice-cold HPLC-grade acetonitrile (1: 3 v/v ratio) in 1.5 mL Eppendorf tubes, followed by vigorous vortexing for 15 s using a Scientific Industries Vortex-Genie 2 at maximum speed and centrifugation at 13,000 × g for 10 min at 4°C using a refrigerated Eppendorf 5424R centrifuge, with the resulting supernatant transferred to new tubes using gel-loading pipette tips. For sample concentration and reconstitution, supernatants were dried in a Thermo Scientific SPD1010 SpeedVac concentrator at 30°C under 50 mTorr vacuum pressure for 4 h, and the dried extracts were reconstituted in 100 μL of 75% methanol/25% water (v/v) containing 0.1% formic acid as an ionization enhancer, followed by vortexing for 15 s and recentrifugation under the same conditions. For quality control, five technical replicates of each sample were prepared, and pooled QC samples were generated by combining equal volumes (10 μL) from all study samples, with five QC replicates analyzed at the beginning of the run, after every 10 experimental samples, and at the end of the sequence to monitor system stability and reproducibility throughout the metabolomic analysis.

Chromatographic separation was performed on a Thermo Scientific UltiMate 3000 RSLCnano system equipped with a Waters ACQUITY UPLC BEH C18 column (50 mm × 2.1 mm, 1.7 μm particle size, 130 Å pore size) maintained at 30 ± 0.5°C, using mobile phases consisting of 0.1% formic acid in ultrapure water (18.2 MΩ·cm, phase A) and 0.1% formic acid in LC-MS grade methanol (phase B) with a gradient program (0–2 min: 5% B; 2–8 min: 5–95% B; 8–10 min: 95% B; 10.1–12 min: 5% B) at a constant flow rate of 0.3 mL/min and 2 μL injection volume in partial loop mode, while mass spectrometric analysis was conducted using a Q Exactive HF-X Hybrid Quadrupole-Orbitrap mass spectrometer with a HESI-II ion source operated at ±3.0 kV spray voltage, 320°C capillary temperature, 300°C probe heater temperature, and sheath/auxiliary gas flows of 30/10 arbitrary units, acquiring data in both positive and negative ionization modes with full MS scans (70,000 resolution at *m*/*z* 200, 100–1,000 *m*/*z* range, 3 × 10^6^ AGC target, 100 ms max injection time) and data-dependent MS/MS scans (17,500 resolution, top 10 precursors, 1.2 *m*/*z* isolation window, stepped 20/40/60 eV NCE, 1 × 10^5^ AGC target, 10 s dynamic exclusion). Raw data were converted using ProteoWizard msConvert (v3.0.20023) with peak picking and intensity threshold filtering, followed by feature detection in XCMS Online (v3.7.1) using CentWave (peakwidth 5–20 s, S/N threshold 10), Obiwarp (0.5 *m*/*z* bin size), and grouping (5 s bandwidth, 50% minimum fraction) algorithms, with quality control based on <30% RSD in QC samples and removal of features detected in <50% of QCs, while compound annotation was performed against HMDB (v4.0) and mzCloud databases requiring <5 ppm mass accuracy, >0.7 MS/MS similarity score, and <0.2 min retention time deviation from standards, with method validation demonstrating <15% intra-day RSD for 90% metabolites, 24-h sample stability at 10°C, 85–115% recovery rates for spiked standards, and established LOD/LOQ values for 50 representative metabolites.

### Statistical analysis of the serum metabolomic data

Mass spectrometry data were processed using Compound Discoverer 3.0 (Thermo Fisher) with a 0.2 min retention time window for peak alignment, QC-based LOESS signal correction for normalization, and removal of features showing ≥50% presence in procedural blanks. Multivariate analysis was performed in SIMCA-P14.1 (Umetrics) using Pareto scaling and log10 transformation for data pretreatment, with model validation through 200 permutation tests (*R*^2^*Y* intercept <0.4) and 7-fold cross-validation (*Q*^2^ > 0.5 threshold for model acceptance). Significant metabolites were selected based on variable importance in projection (VIP) scores >1 from OPLS-DA models, Welch’s *t*-test *p*-values <0.05 after Benjamini–Hochberg false discovery rate correction, and fold changes >1.8 between experimental groups. Metabolite and metabolic pathway analyses were performed via MetaboAnalyst 6.0^1^ and the KEGG database.^2^ Metabolite traceability analysis was conducted via MetOrigin 2.0^3^ (Yu et al., 2024).

### Statistical analysis

All processed data, unless explicitly stated, were loaded into R and Perl for analysis and visualization. Data processing was conducted via SPSS 27.0 statistical software, and measurements that were normally distributed are presented as the means ± standard deviations (X̅ ± S). To assess the significance of differences, *t*-tests were performed with GraphPad Prism 9 software. Significance levels are denoted as follows: ^*^p ≤ 0.05, ^**^p ≤ 0.01, ^***^p ≤ 0.001, and ^****^p ≤ 0.0001, with *p*-values serving as the criterion for significance determination.

## Results

### Significant fluctuations in the rumen microbial community structure between the lactation and dry periods

**Figure 1:**
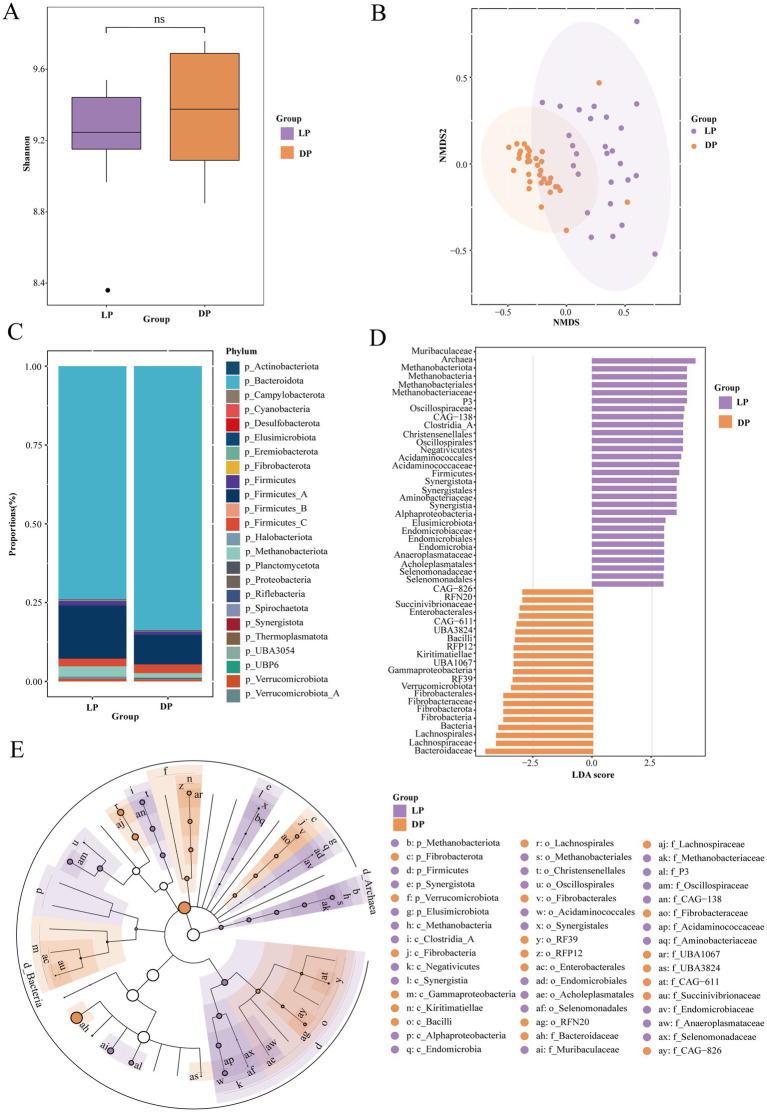
Microbial composition of the rumen in buffaloes during lactation (*n* = 29) and dry periods (*n* = 33). **(A)** Alpha-diversity of rumen microorganisms in buffaloes during lactation and dry periods. **(B)** Analysis of the β-diversity of rumen microorganisms in the lactation and dry period buffaloes via NMDS. **(C)** Percentage stacked plots of phylum levels in the lactation and dry period buffaloes. **(D)** Histogram of LDA scores of different microbial abundances between the microorganisms of the lactation and dry period buffaloes demonstrating the species that differed at LDA scores >2, *p* < 0.05, and significantly different biomarkers, with the length of the histograms representing the magnitude of influence of the significantly different species. **(E)** Evolutionary branching diagram of species differing between the lactation and dry period buffaloes, with circles radiating inwards to outwards representing taxonomic levels from the phylum to the genus level. Each small circle at a different taxonomic level represents a taxon at that level, and the size of the circle diameter is proportional to the size of the relative abundance; species with no significant differences are uniformly colored white, purple nodes indicate microbial taxa that play an important role in the lactation buffalo group, and yellow nodes indicate microbial taxa that play an important role in the dry group.

To identify potential differences in the structural composition of the rumen microbiota between the lactation and dry periods, we collected 29 rumen fluid samples from buffaloes in the lactation period (LP) and 33 from buffaloes in the dry period (DP) for metagenomic analysis (Supplementary Figure S1). We used α-diversity and β-diversity as metrics for analysis, but alpha diversity did not differ significantly between the two groups (Figure 1A). However, β-diversity analysis revealed significant differences in the rumen microbiota between the lactation and dry periods (*p* < 0.001, Figure 1B). Additionally, the distances between the lactation samples were more dispersed, whereas those between the dry samples were more clustered, suggesting that the microbial community in buffaloes shows greater functional diversity during lactation.

At the phylum level (Figure 1C), *Bacteroidota* and *Firmicutes* were the dominant phyla. Notably, the abundances of *Firmicutes_A* (LP: 16.88%, DP: 9.45%) and *Bacteroidetes* (LP: 73.93%, DP: 83.74%) changed significantly between the two periods. Other phyla showing notable differences included *Methanobacteriota* (LP: 3.30%, DP: 1.47%) and *Verrucomicrobiota* (LP: 0.73%, DP: 0.44%). To further elucidate the differences in the rumen microbial communities between lactating buffaloes and dry buffaloes and to identify specific biomarkers, we conducted linear discriminant analysis (LDA) and linear discriminant analysis effect size (LEfSe). We identified 50 distinct organisms that met the significance thresholds (|LDA| ≥2.5, *p* < 0.05; Figure 1D). LEfSe provided a visual representation of the dominant microbial groups from the phylum to family levels via representative structural branching maps (Figure 1E). The bacterial families most abundant during lactation included *Acidaminococcaceae*, *Muribaculaceae*, *Methanobacteriaceae*, *Endomicrobiaceae*, *Anaeroplasmataceae*, and *Selenomonadaceae*. In contrast, the bacterial families that were more abundant during the dry period included *Succinivibrionaceae*, *Kiritimatiellae*, *Fibrobacteraceae*, *Lachnospiraceae*, and *Bacteroidaceae*.

In summary, the richness and evenness of rumen microbiota in buffaloes are similar between the lactation and dry periods, but there are significant differences in species composition, indicating the potential impact of host status on feed nutrient utilization efficiency.

### Buffalo rumen microorganisms form different groups between the lactation and dry periods

**Figure 2:**
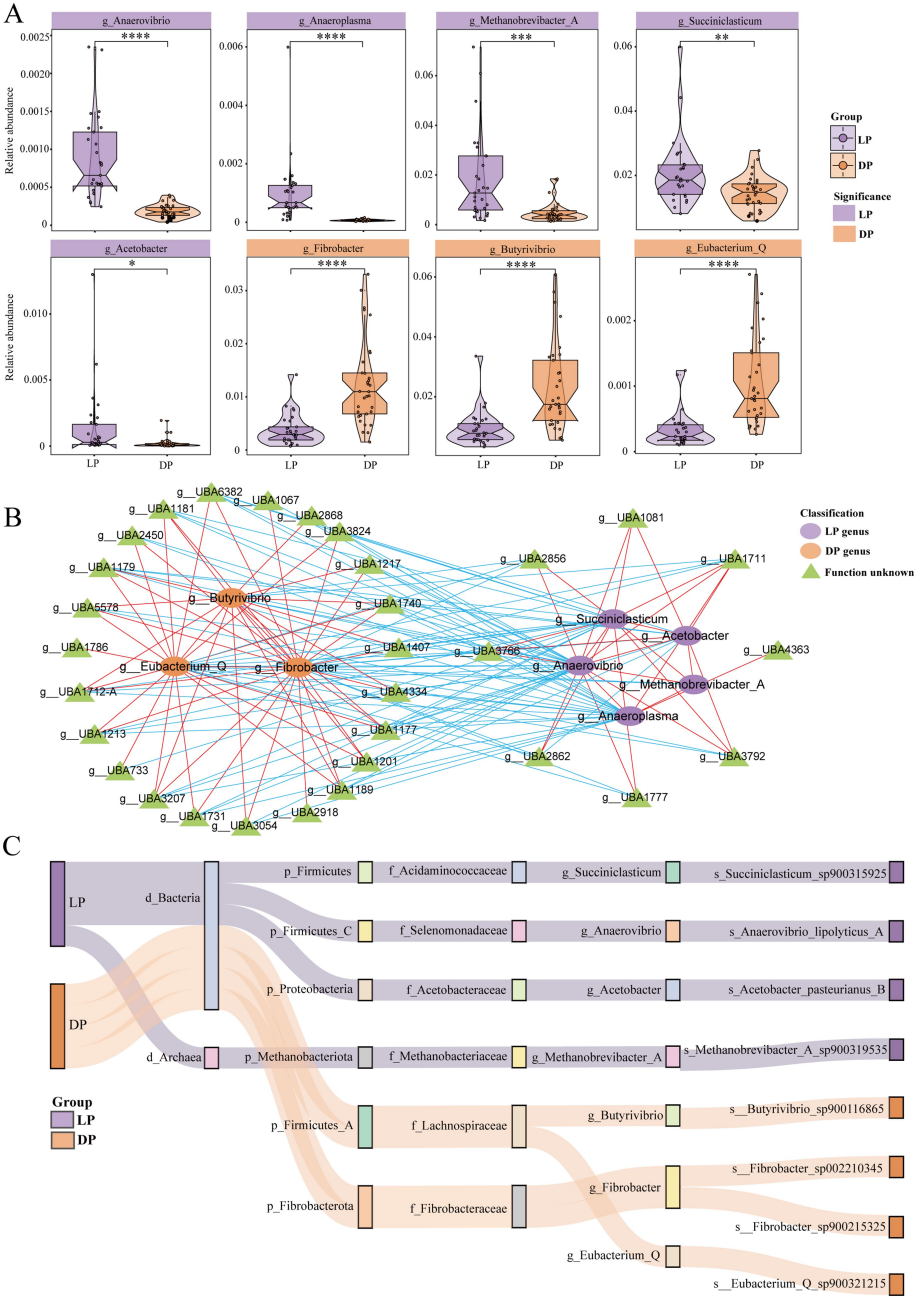
Differences and correlations between bacterial genera in buffaloes during lactation (*n* = 29) and dry periods (*n* = 33). **(A)** Violin plots for genera with significant differences between lactating buffaloes and dry buffaloes, with purple representing the lactation group and yellow representing the dry group; the statistically significant marker * represents * for *p* ≤ 0.05; ** for *p* ≤ 0.01; *** for *p* ≤ 0.001; and **** for *p* ≤ 0.0001. **(B)** Network diagram of the correlations of different genera in lactating buffaloes and dry buffaloes. The more abundant genera in the lactation period are purple nodes, the more abundant genera in the dry period are yellow nodes, and the functionally unknown genera are green nodes; red lines indicate positive correlations, and blue lines indicate negative correlations. **(C)** Sankey diagram of microbial classification. Each column of nodes from left to right represents classification, phylum, family, genus, or species. The purple line represents significant enrichment during the lactation period, and the yellow line represents significant enrichment during the dry lactation period.

To investigate potential functionally distinct bacterial genera, we further examined the differences in the distribution of the rumen microbiota across the lactation period in buffaloes. At the genus level (Figure 2A), bacterial genera with higher abundances in the lactation period group included *Anaerovibrio* (*p* < 0.0001), which is linked to lipid hydrolysis (Zhu et al., 2022). *Anaeroplasma* (*p* < 0.0001) can contribute to the production of propionic acid (Choi et al., 2022). *Acetobacter* (*p* < 0.05) is capable of producing acetate during rumen fermentation (Kim et al., 2022). *Succiniclasticum* (*p* < 0.01) converts succinate to propionate (van Gylswyk, 1995). *Methanobrevibacter_A* (*p* < 0.001) has been associated with methane production in ruminant studies (Kelly et al., 2016). The bacterial genera with relatively high abundances in the dry period included *Butyrivibrio* (*p* < 0.001), *Fibrobacter* (*p* < 0.001) and *Eubacterium_Q* (*p* < 0.001). The former two are related to the degradation of lignocellulose, and the latter is related to the synthesis of butyric acid (Mukherjee et al., 2020; Yang et al., 2021). In addition, we observed that the genera *Klebsiella* (*p* < 0.05), *Enterobacter* (*p* < 0.05), and *Comamonas* (*p* < 0.001), were significantly more abundant during the dry period (Supplementary Figure S2). These genera are recognized as opportunistic pathogens and have been associated with bovine mastitis as well as gastrointestinal disorders (Isaac et al., 2025; Massé et al., 2020).

To explore the interactions between bacterial genera across different periods, we conducted a correlation analysis (Figure 2B). During the dry period, positive correlations were observed among *Butyrivibrio*, *Eubacterium_Q*, and *Fibrobacter*, which were also associated with 23 unidentified bacterial genera with similar functions. Conversely, in the lactation group, positive correlations were detected among *Anaerovibrio*, *Anaeroplasma*, *Acetobacter*, *Succiniclasticum*, and *Methanobrevibacter_A*, along with eight unknown bacterial genera that share similar functions. We further performed species-level identification of the eight bacterial genera mentioned above (Figure 2C). The results indicated that *S*. sp. *900315925*, *A. lipolyticus s_A*, *A. pasteurianus_B*, and *M. A* sp. *900319535* were detectable during the lactation period. In contrast, *B.* sp. *900116865* and *E_Q* sp. *900321215* were identifiable during the dry period. Notably, *g_Fibrobacter* could identify two bacterial strains: *F.* sp. *002210345* and *F.* sp. *900215325*.

In summary, the rumen bacterial genera enriched during the lactation period in buffaloes were more efficient at producing methane, short-chain fatty acids, and other metabolites. In contrast, the genera enriched during the dry period were associated primarily with increased cellulose digestibility.

### Diversity of functions performed by rumen microorganisms in buffaloes between lactation and dry periods

**Figure 3:**
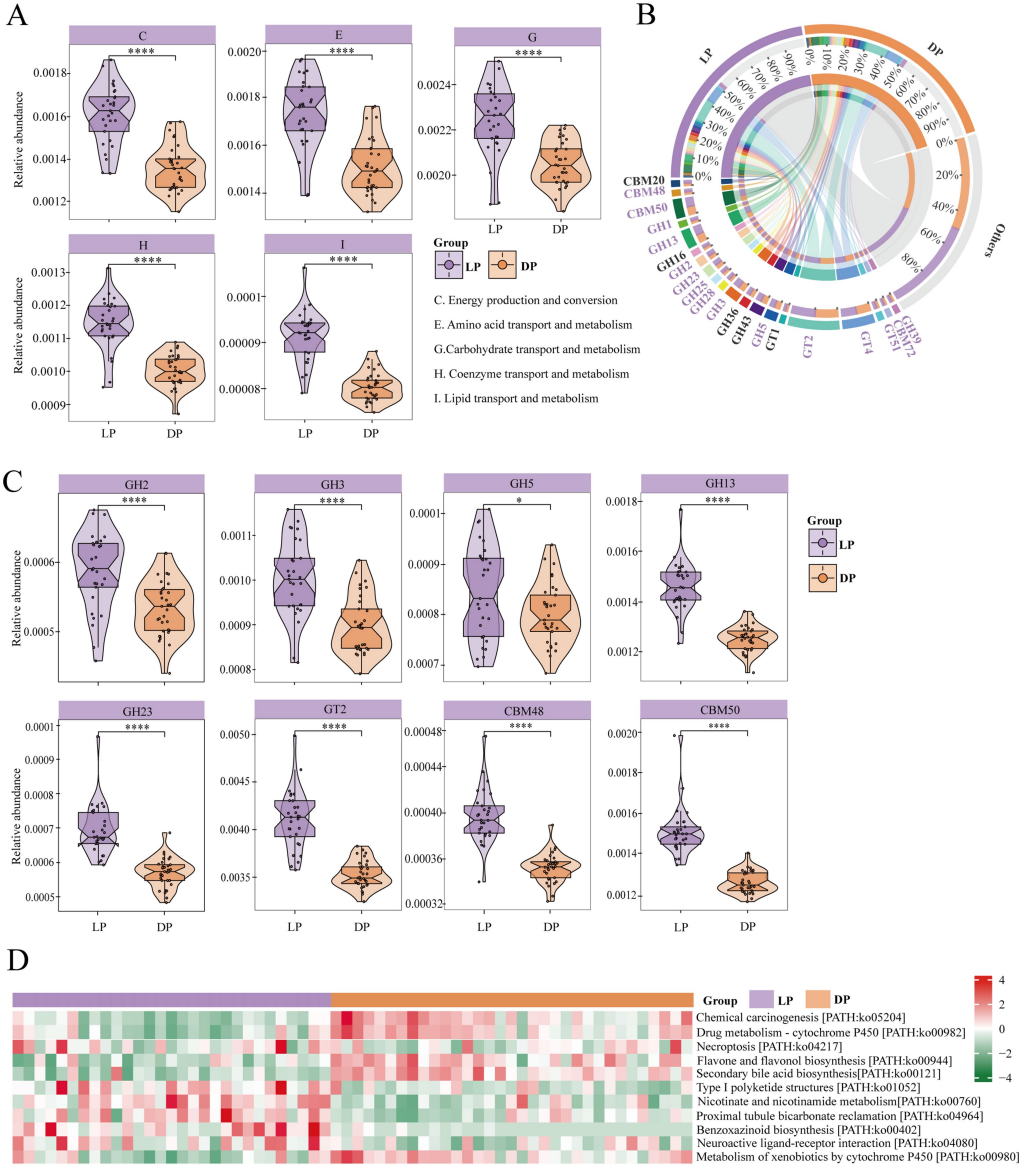
Functional composition and differential analysis of rumen microbial communities in buffaloes during lactation (*n* = 29) and dry periods (*n* = 33). **(A)** Violin plots for COGs with significant differences between lactating buffaloes and dry buffaloes, with purple representing the lactation group and yellow representing the dry group; the statistically significant marker **** for *p* ≤ 0.0001. **(B)** CAZymes with a relative abundance >1% in buffalo during lactation and dry periods. Purple markings indicate high abundance during the lactation period, and black markings indicate high abundance during the dry lactation period. **(C)** Violin plots for CAZymes with significant differences between lactating buffaloes and dry buffaloes, with purple representing the lactation group and yellow representing the dry group; the statistically significant marker * for *p* ≤ 0.05 and **** for *p* ≤ 0.0001. **(D)** Heatmap of three-level pathways in buffalo during lactation and dry periods. The red modules represent positive correlations, and the green modules represent negative correlations.

To explore the functions of the identified microbiota, we functionally annotated the predicted acquired genes via the COG, CAZyme, and KEGG databases. In the COG database (Figure 3A), the modules significantly enriched in the lactation period included C-Energy Production and Conversion (*p* < 0.0001), reflecting increased microbial ATP generation to meet the high metabolic demands of lactation; E-amino acid transport and metabolism (*p* < 0.0001), indicating enhanced protein turnover to supply essential amino acids for milk protein synthesis; G-carbohydrate transport and metabolism (*p* < 0.0001), suggesting upregulated polysaccharide degradation to provide glucose precursors for lactose production; H-coenzyme transport and metabolism (*p* < 0.0001), potentially supporting vitamin and cofactor biosynthesis for enzymatic reactions; and I-lipid transport and metabolism (*p* < 0.0001), likely facilitating microbial lipid metabolism to contribute to milk fat synthesis. These findings suggest that the rumen microbiota in buffaloes during lactation may increase the conversion of feed into metabolites such as amino acids and energy. Additionally, certain bacteria may have the ability to synthesize or activate metabolic pathways related to coenzymes, thereby supporting milk synthesis.

Next, we examined the differences in enzyme activity between microorganisms in the two periods and identified the dominant enzymes with a relative abundance greater than 1% (Figure 3B).

The abundance of enzymes utilized by rumen microorganisms in lactating buffaloes was notably greater than that in healthy buffaloes. Among them (Figure 3C), the glycoside hydrolase family included GH2 (*p* < 0.0001), GH3 (*p* < 0.0001), GH5 (*p* < 0.05), and GH13 (*p* < 0.0001). The significant enrichment of these GH families, particularly GH13 (α-amylase), suggests enhanced capacity for starch and glycogen breakdown (Janeček and Zámocká, 2020), which may provide essential glucose precursors for lactose synthesis during lactation. The carbohydrate-binding module family includes CBM48 (*p* < 0.0001) and CBM50 (*p* < 0.001), whose increased abundance likely improves microbial access to complex carbohydrates by enhancing enzyme-substrate interactions (Renaud et al., 2023; Zhang et al., 2022), whereas the glycosyltransferase family includes GT2 (*p* < 0.0001). This GT2 enrichment is particularly noteworthy as it includes enzymes involved in lactose biosynthesis (Lampugnani et al., 2024)., potentially indicating microbial adaptation to support the host’s milk production demands. GH2, GH3, GH5, and GH23 all contain β-glucosidase, an enzyme capable of hydrolysing glucosides into free glucose (Wang et al., 2021), suggesting increased microbial capacity to liberate glucose from plant biomass, which could contribute to the energy requirements of lactation. Collectively, these CAZyme profile changes demonstrate a coordinated microbial adaptation toward enhanced carbohydrate metabolism that aligns with the increased nutritional demands of lactating buffaloes.

We further identified the metabolic pathways of rumen microbes. At the tertiary pathway level of KEGG, 11 differential metabolic pathways were identified (Figure 3D). The results revealed that the pathways significantly enriched during lactation included PATH: ko01052, PATH: ko00402, PATH: ko04080, PATH: ko04964, PATH: ko00760, and PATH: ko00980 (*p* < 0.05). Notably, ko04080 is a neurotransmitter signalling pathway that regulates nervous system function, influencing animal mood and feed intake. ko00760 is involved in NAD + synthesis, which supports cellular energy metabolism and repair. The pathways significantly enriched during the dry period included PATH: ko05204, PATH: ko00982, PATH: ko04217, PATH: ko00944, and PATH: ko00121 (*p* < 0.05). The first four pathways are closely associated with inflammation and immune responses. In contrast, PATH ko00121 is involved in the conversion of primary bile acids to secondary bile acids, which may increase the digestive capacity of buffaloes.

Overall, the rumen microbial function in lactating buffaloes appears to be more effective at promoting feed intake, utilizing a range of enzymes to produce nutrients relevant to milk synthesis. In contrast, during the dry period, rumen microorganisms in buffaloes seem to prioritize enhancing immune function and secreting bile acids to maintain intestinal health.

### Significant fluctuations in serum metabolites between the lactation and dry periods

**Figure 4:**
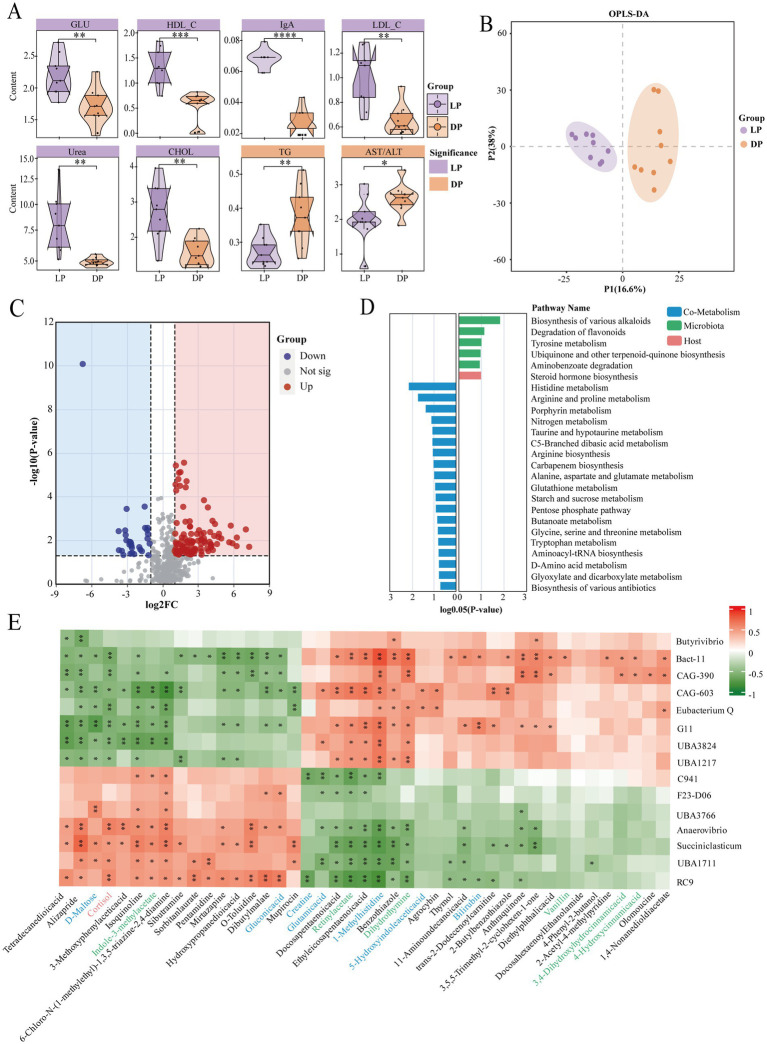
Analysis of serum biochemical indices and metabolite composition in buffaloes during lactation (*n* = 9) and dry periods (*n* = 9). **(A)** Violin plots of serum biochemical indicators in lactating buffaloes and dry buffaloes. The color blocks where the names of the indicators are located are purple for significantly higher values in lactating buffaloes, yellow for significantly higher values in dry buffaloes, and the statistically significant markers * stand for * for *p* ≤ 0.05, ** for *p* ≤ 0.01, *** for *p* ≤ 0.001, and **** for *p* ≤ 0.0001. **(B)** OPLS-DA plots of serum metabolites in positive and negative ion modes. **(C)** Volcano plot of serum metabolites in lactating buffaloes and dry buffaloes (log2FC = 1, *p* = 0.05); red dots are upregulated, blue dots are downregulated, and gray dots indicate no difference. **(D)** Metabolic pathway enrichment analysis of serum differentially abundant metabolites in lactating buffaloes and dry buffaloes. The blue color block represents cometabolism, the green color block represents microbial metabolism, and the orange color block represents host metabolism. **(E)** Spearman correlation between microbial genera and metabolites. The blue font represents cometabolites, the green font represents microbial metabolites, and the orange font represents host metabolites.

To explore differences in serum composition, we measured a total of 20 serum biochemical indices in buffaloes during the lactation and dry periods (Supplementary Figure S3). Among these indices, 8 indices were significantly different between the two groups (Figure 4A). In the lactation group, six indicators were significantly greater: high-density lipoprotein (HDL, *p* < 0.001), low-density lipoprotein (LDL, *p* < 0.01), glucose (Glu, *p* < 0.01), immunoglobulin A (IgA, *p* < 0.0001), cholesterol (CHOL, *p* < 0.01), and urea nitrogen (Urea, *p* < 0.01). Two indicators were significantly greater during the dry period: triglycerides (TG, *p* < 0.01) and the ratio of glutamic oxaloacetic transaminase to glutamic pyruvic transaminase (AST/ALT, *p* < 0.05).

We conducted nontargeted metabolomics analysis using the UPLC–MS/MS platform and identified 307 metabolites in both positive and negative ion modes. To provide a comprehensive overview, we included an OPLS-DA score plot to illustrate overall group separation (Figure 4B) and retained individual visualizations of metabolite profiles for each ionization mode (Supplementary Figure S4 for positive mode; Supplementary Figure S5 for negative mode). Further screening revealed 108 upregulated and 48 downregulated differentially abundant metabolites between the two groups (|log2FC| >1, *p* = 0.05; Figure 4C). A traceability analysis of these metabolites was conducted via MetOrigin 2.0 software to determine their sources. The results (Supplementary Figure S6) indicate that most metabolites originate from food, the host, and the microbiota. Through pathway analysis of these 156 significantly different metabolites, we identified 5 enriched microbial metabolic pathways, 1 enriched host metabolic pathway, and 19 enriched cometabolic pathways (Figure 4D). Notably, pathways such as biosynthesis of various alkaloids, flavonoid degradation, tyrosine metabolism, steroid hormone biosynthesis, histidine metabolism, arginine and proline metabolism, porphyrin metabolism, nitrogen metabolism, taurine and hypotaurine metabolism, C5-branched dibasic acid metabolism, arginine biosynthesis, and carbapenem biosynthesis significantly differed (*p* < 0.05).

Spearman correlation analysis was performed on the differentially abundant metabolites and rumen microbial genera to further explore the interactions between metabolites and microorganisms (Figure 4E). The results revealed that two microbial genera, *Anaerovibrio* and *Succiniclasticum*, which had significantly greater abundances during lactation, were positively correlated with 17 metabolites. Additionally, *UBA3766* and *UBA1711* were positively correlated with these metabolites, supporting our previous speculation regarding the potential functions of these unknown bacterial genera. However, *Bacteroides*, *Eubacterium_Q*, *UBA3824*, and *UBA1217*, which had significantly greater abundances during the dry period, were positively correlated with 26 metabolites. According to the traceability analysis, seven of the metabolites related to these microorganisms are derived from cometabolism, six from the microbiota, and one from the host.

In summary, host metabolism reflects the associations of both groups of buffalo with lipid synthesis and utilization, carbohydrate synthesis, and immune protein synthesis.

### Joint analysis of the rumen microbiota and serum metabolites in buffaloes during lactation and dry periods

**Figure 5:**
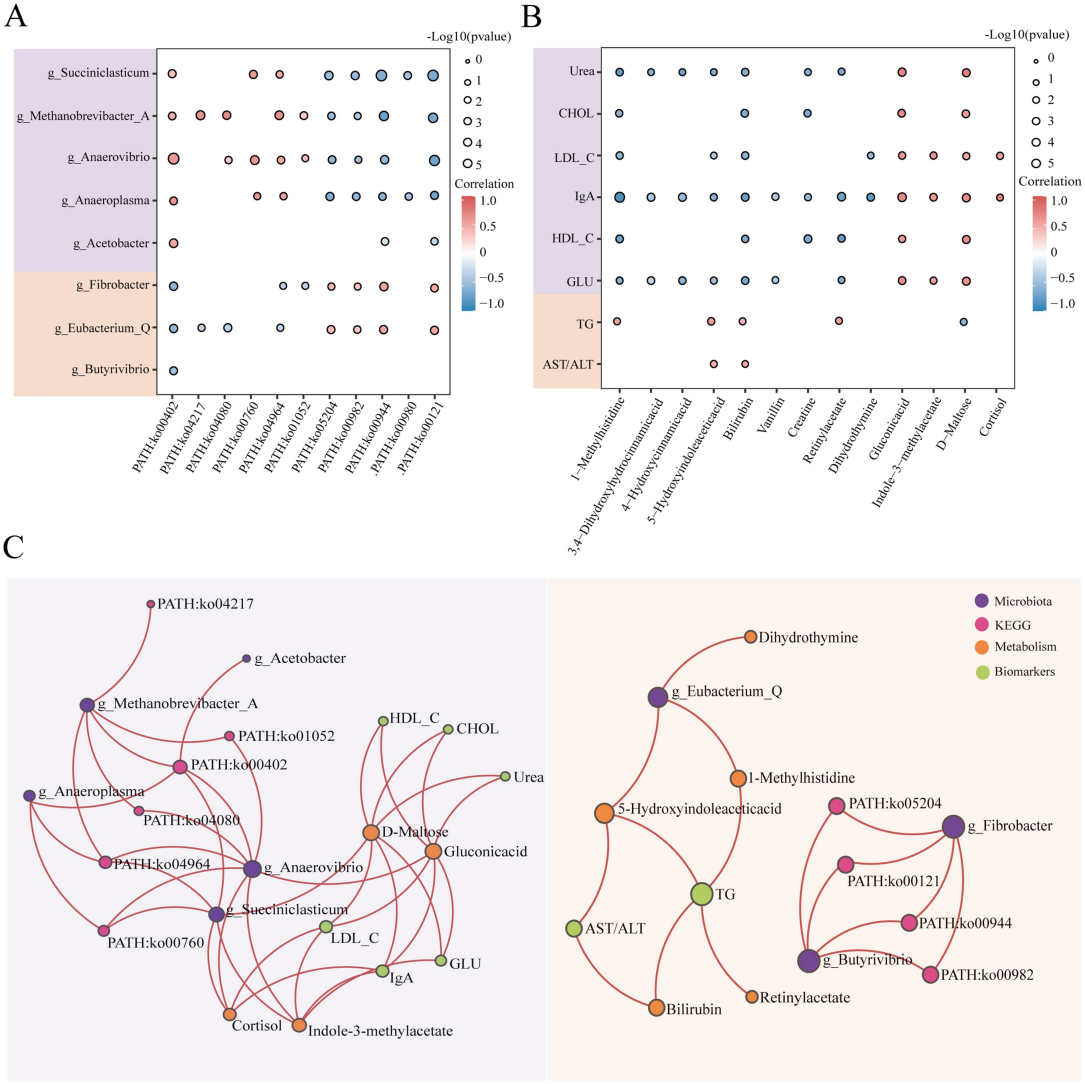
Multiomics joint analysis of buffaloes during lactation (*n* = 9) and dry periods (*n* = 9). **(A)** Spearman correlation between ionic differential microbial genera and KEGG third-level pathways. The purple module represents bacterial genera with significantly greater abundance during the lactation period, and the yellow module represents bacterial genera with significantly greater abundance during the dry lactation period. The size of the bubbles represents the level of significance; the smaller the *p*-value is, the larger the bubbles. Red represents a positive correlation, and blue represents a negative correlation. **(B)** Spearman correlation between landmark differentially abundant metabolites and biochemical indicators. The purple module represents biochemical indicators with significantly greater abundance during the lactation period, and the yellow module represents biochemical indicators with significantly greater abundance during the dry lactation period. The size of the bubbles represents the level of significance; the smaller the *p*-value is, the larger the bubbles. Red represents a positive correlation, and blue represents a negative correlation. **(C)** Positive correlation network diagram of microbial genera, KEGG pathways, metabolites and biochemical indicators (correlation >0.5, *p* < 0.05). Purple represents microorganisms, pink represents KEGG, yellow represents metabolites, and green represents biochemical indicators.

To further investigate the relationships between characteristic microbiota and their functional roles during the lactating and dry periods, as well as the connections between key serum metabolites and biochemical indicators, we conducted a Spearman correlation analysis. From this joint analysis (Figure 5A), we observed that five microbial genera, which were significantly more abundant during lactation, were positively correlated with PATH: ko00402 and negatively correlated with PATH: ko00944 and PATH: ko00121. Conversely, three microbial genera with relatively high relative abundances during the dry period presented a significant negative correlation with PATH: ko00402. Notably, *g_Fibrobacter* and *g_Eubacterium_Q* were positively correlated with PATH: ko00944 and PATH: ko00121. In the joint analysis of biochemical indicators and metabolites (Figure 5B), gluconic acid and D-maltose were significantly positively correlated with six biochemical indicators that presented significantly greater relative abundances during lactation. Bilirubin and 5-hydroxyindoleacetic acid were significantly positively correlated with TG and AST/ALT, which were more abundant during the dry period. We conducted Spearman correlation analyses between biochemical parameters and both CAZy families and COG functional categories. The results revealed that all six CAZy enzyme families exhibited negative correlations with triglyceride levels, with CBM50 showing a highly significant positive correlation with glucose concentration (Supplementary Figure S7). Similarly, COG categories C, E, G, H, and I were negatively correlated with triglycerides, while category I displayed a positive association with glucose levels (Supplementary Figure S7). These findings suggest that specific functional enzymes and gene categories may be involved in host lipid and glucose metabolism regulation.

We also plotted positive correlations between the lactation and dry periods to predict the connections between rumen microorganisms and host metabolism (Figure 5C). During lactation, *g_Anaerovibrio* may produce gluconic acid through co-metabolism, whereas *g_Succiniclasticum* may produce D-Maltose through co-metabolism or generate Indole-3-methylacetate through microbial metabolism. Both D-maltose and gluconic acid may participate in the synthesis of various biochemical indicators. During the dry period, *g_Eubacterium_Q* may produce 1-methylhistidine and 5-hydroxyindoleacetic acid through co-metabolism, and these two metabolites could be involved in TG synthesis.

## Discussion

Through combined multiomics analysis, our study identified the taxonomic characteristics of rumen microorganisms, their functions, and their interactions with host metabolism, all of which are associated with changes in the physiological state of the buffaloes.

### Host-driven rumen microbiome remodeling: compositional and functional shifts

Although the dietary composition differed between lactation and dry periods, we observed no significant differences in alpha diversity indices (richness and evenness) of the rumen microbiota. This stability in α-diversity may be attributed to the resilience and ecological buffering capacity of core microbial taxa, which are known to maintain compositional equilibrium under varying environmental and nutritional conditions (Fassarella et al., 2021). However, significant differences were observed in the key species composition and function of rumen microbiota between lactation and dry periods, which may be attributed to differences in host metabolic requirements, leading to adaptive changes in microbial community function. During lactation, the bovine gastrointestinal microbiome plays a crucial role in breaking down indigestible plant polysaccharides and providing precursors for milk synthesis, including amino acids, fatty acids, and glycoheterophilic compounds (Monteiro et al., 2022). Consequently, rumen microorganisms in buffaloes exhibit more complex and varied functions during lactation than during the dry period. In terms of composition, the predominant phyla in the buffalo rumen were *Bacteroidota* and *Firmicutes*, which is consistent with previous research. *Bacteroidetes* contribute primarily to carbohydrate degradation and polysaccharide utilization (Grondin et al., 2017), whereas *Firmicutes* are involved mainly in fibre degradation (Barczynska et al., 2016). Furthermore, the *Firmicutes/Bacteroidetes* (F/B) ratio is relatively high during lactation, which enhances the capacity of buffalo to extract nutrients from feed (Tong et al., 2022). Several of the bacterial genera enriched in lactating buffaloes, such as *Anaerovibrio*, S*ucciniclasticum*, and *Methanobrevibacter*, have also been reported as key taxa in the rumen of dairy cows and goats during lactation. *Anaerovibrio* and *Succiniclasticum* are associated with lipid and propionate metabolism, respectively, and their abundance has been linked to higher energy demands during milk production in dairy cows (Naing et al., 2025). Similarly, *Methanobrevibacter* is a dominant archaeal genus consistently observed in lactating ruminants, playing a central role in hydrogenotrophic methanogenesis (Shinkai et al., 2024). In contrast, genera such as Fibrobacter and Butyrivibrio, which were more abundant in buffaloes during the dry period, have also been reported as dominant fiber-degrading taxa in Simmental steers, suggesting a similar rumen microbial adaptation to high-fiber diets (Wang et al., 2015). These comparisons suggest that the microbial shifts observed in buffaloes may reflect conserved rumen microbial adaptations across ruminant species in response to physiological state and dietary changes.

In addition to core functional taxa, several potential pathogens including *Klebsiella*, *Enterobacter*, and *Comamonas* were significantly enriched during the dry period. These genera are associated with bovine mastitis and gastrointestinal disorders, suggesting potential risks to animal health and milk safety. The findings are consistent with recent concerns raised by Tsakmakidis et al. (2024) who emphasized the risk of pathogenic bacterial transmission from the ruminant gastrointestinal tract to milk, potentially resulting in public health hazards, particularly in systems lacking strict hygienic controls. Although the rumen is not a direct source of milk contamination, the possibility of microbial translocation via hematogenous spread or ascending routes, particularly under conditions of physiological stress or immunosuppression, cannot be excluded (Zinicola et al., 2015). The increased prevalence of these opportunistic taxa during the dry period, characterized by immune and metabolic shifts, highlights a potential risk. These findings emphasize the need to monitor not only the functional dynamics of the rumen microbiota but also the presence of potentially pathogenic organisms that may compromise milk quality and safety.

In this study, we observed that several glycoside hydrolases in the CAZyme family were significantly more abundant during the lactation period than during the dry period. These include GH2 (β-glucosidase), GH3 (β-glucosidase), and GH5 (polysaccharide hydrolase), which are involved in cellulose degradation. These findings suggest that lactating buffaloes have increased sugar decomposition and absorption to meet their high carbohydrate demands (Chang et al., 2018). Additionally, GH13 (α-amylase) and GH23 (lysozyme) are enzymes with broad-spectrum hydrolytic activity. The increased abundance of these bacteria during lactation may reflect the greater breakdown of carbohydrates and polysaccharides in the intestine, indicating an increase in nutrient digestion by the microbiota during this period (van Wyk et al., 2017).

In the functional annotation of bacterial genera, the abundances of PATH: ko00760, PATH: ko04964, and PATH: ko04080 were significantly greater during lactation than during the dry period. PATH: ko00760 involves niacin (vitamin B3), which is a precursor of NAD^+^, a molecule essential for various physiological processes, such as cellular energy metabolism, DNA repair, and cell signalling. This may be linked to the repair of mammary gland cells in lactating buffaloes (Orlandi et al., 2020). PATH: ko04964 is related to bicarbonate reabsorption in the proximal tubule, which may be particularly important in lactating buffaloes, as they lose substantial amounts of water and electrolytes during milk secretion. This pathway helps maintain acid–base and electrolyte balance (Lu et al., 2021). Finally, PATH: ko04080 plays a crucial role in regulating mammary gland secretory function, milk synthesis, and endocrine signalling during lactation through neuroactive ligands (Xu et al., 2023).

### Serum metabolomics reveals lactation specific metabolic reprogramming

In the serum metabolomics analysis of the two groups of buffaloes, the biosynthesis of various alkaloids was notably more pronounced in lactating buffaloes. These alkaloids function as protective agents against infections and oxidative damage, thereby enhancing the immune capacity of buffaloes during lactation (Manogaran et al., 2023). Tyrosine metabolism plays a pivotal role in physiological regulation, as tyrosine serves as a key precursor for the synthesis of thyroid hormones triiodothyronine (T3) and thyroxine (T4). These hormones are essential for maintaining energy homeostasis, thermoregulation, and growth. In lactating buffaloes, elevated tyrosine metabolism may reflect increased thyroid hormone activity to meet the heightened energy demands associated with milk production (Basolo et al., 2022). The observed differences in the histidine metabolism pathways may be attributed to the conversion of histidine into histamine, a neurotransmitter that enhances feeding efficiency, which explains the increased feed intake observed in lactating buffaloes during this period (Moro et al., 2020). C5-branched dibasic acid metabolism is a key component of protein synthesis and is potentially involved in the synthesis of milk proteins during lactation (Wu et al., 2024). Arginine and proline metabolism and nitrogen metabolism are integral parts of the urea cycle and play direct roles in nitrogen excretion and metabolism. During lactation, buffaloes must synthesize milk components, thereby increasing their utilization of amino acids and accelerating nitrogen metabolism (Wu et al., 2022). Furthermore, the proportion of concentrated feed in the diet of lactating buffaloes was greater than that during the dry period, which further contributed to the increased nitrogen supply. Overall, these metabolic pathway alterations suggest that lactating buffaloes undergo complex physiological adaptations in energy metabolism, immune regulation, and milk component synthesis.

In addition to fluctuations in serum metabolites, we also investigated whether serum biochemical parameters are associated with host-driven functional changes in rumen microbiota. Correlation analysis between serum biochemical indices and COG and CAZy functions revealed that multiple CAZy enzyme families were significantly negatively correlated with serum triglyceride levels. Notably, CBM50 showed a significant positive correlation with elevated blood glucose concentration. Within the COG functional categories, category I (related to carbohydrate transport and metabolism) was also positively correlated with blood glucose levels. These findings suggest that under the high energy demand during lactation, the rumen microbiota may indirectly contribute to host lipid mobilization and glucose supply by enhancing carbohydrate degradation and transport capabilities, thereby supporting the mammary gland’s substrate requirements for lactose synthesis (Mao et al., 2015). Similar phenomena have been observed in studies on goats, where researchers noted an increased carbohydrate metabolic capacity of rumen microbes during lactation to adapt to the metabolic pressure imposed by milk production (Han et al., 2021). Therefore, our results further support the concept of host metabolic status regulating microbial functional expression, indicating a possible feedback mechanism between rumen microbial ecology and host metabolic regulation.

### Cross-talk between rumen microbes and host metabolites

**Figure 6:**
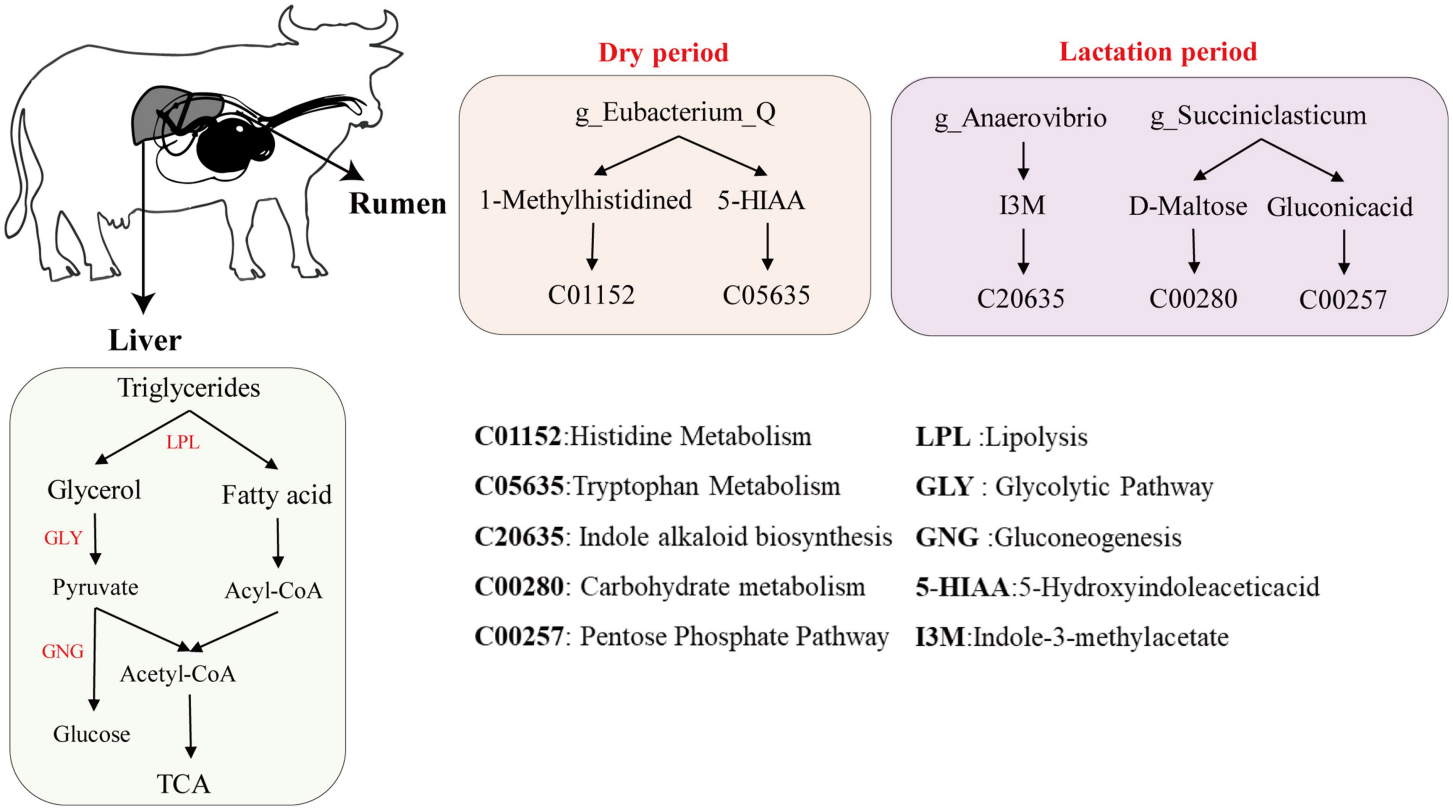
Diagram of the mechanism of hepatic metabolism and synthesis of milk components in buffaloes during lactation and dry periods.

Through comprehensive analysis via metagenomics and serum metabolomics, we investigated the interaction mechanism between rumen bacteria and metabolites in buffalo during the lactation and dry periods (Figure 6). During lactation, the characteristic differential microbial genera in the rumen, such as *g_Anaerovibrio*, may produce the metabolite Indole-3-methylacetate, and *g_Succiniclasticum* may produce D-maltose and gluconic acid. Indole-3-methylacetate (I3M, C20635), a microbial metabolite, may produce indole compounds and participate in indole alkaloid biosynthesis (Nutaratat et al., 2016). D-Maltose (C00208), a disaccharide, is hydrolysed into two glucose molecules by maltase in the maltose metabolism pathway, thereby participating in glycolysis and gluconeogenesis carbohydrate metabolism (Liang et al., 2013). Gluconic acid (C00257), an important metabolic intermediate in the pentose phosphate pathway, contributes to the generation of NADPH, which is a crucial coenzyme in numerous biosynthetic processes (Phégnon et al., 2024). These findings suggest that the interactions between rumen microbial genera and the host during lactation may promote energy metabolism pathways in the body. This phenomenon aligns with findings from studies on lactating dairy cows versus dry dairy cows (Morris and Kononoff, 2021). During the dry period, the characteristic genus *g_Eubacterium_Q* in the rumen may interact with the host to produce the metabolites 1-methylhistidine and 5-hydroxyindoleacetic acid. 1-Methylhistidine (C01152), a derivative of histidine, is recognized as a biomarker of muscle metabolism (Kusy et al., 2024). Interestingly, studies on the proteomics of dairy cows during the dry period suggest that 1-methylhistidine may also be associated with colostrum quality (Siachos et al., 2024). 5-Hydroxyindoleacetic acid (5-HIAA, C05635), a product of the 5-hydroxytryptophan (5-HTP) metabolic pathway, is commonly referred to as serotonin. Serotonin is involved in neurotransmission and plays a role in regulating mood and sleep (Fila et al., 2024). Therefore, we speculate that during the dry period, buffaloes may allocate nutrients to facilitate body recovery and alleviate stress.

We integrated biochemical markers as phenotypic data to further investigate the underlying mechanisms of hepatic metabolism during milk synthesis in buffaloes (Figure 6). These results suggest that during lactation, cholesterol is transported to the liver via low-density lipoprotein (LDL) and subsequently converted into bile acids through a series of enzymatic reactions (Semova et al., 2022). This transformation is central to liver–gut interactions, whereby the liver releases bile acids and antimicrobial agents including primary bile acids, immunoglobulin A, and angiogenin into the intestinal lumen, thereby promoting microbial homeostasis and restraining bacterial overgrowth (Nishida et al., 2020). Biochemical analysis revealed that the serum cholesterol and IgA levels in lactating buffaloes were significantly greater than those in dry buffaloes, further supporting this hypothesis. In terms of lipid metabolism, triglycerides are initially hydrolysed in the liver to glycerol and fatty acids. Glycerol enters the liver and can be converted to pyruvate through glycolysis (GLY). Pyruvate is subsequently converted to acetyl-CoA by pyruvate dehydrogenase and enters the tricarboxylic acid (TCA) cycle, providing energy for milk production (Perry et al., 2020). Additionally, pyruvate can be converted to glucose via gluconeogenesis (GNG), and glucose is transported to the mammary gland for lactose synthesis (Piantoni and VandeHaar, 2023). Fatty acids entering the liver undergo β-oxidation to generate acetyl-CoA, which enters the TCA cycle for further metabolism, supplying energy (Tahri-Joutey et al., 2021). Free fatty acids may also be transported through the bloodstream to the mammary gland for milk fat synthesis (Singh et al., 2023). In milk protein synthesis, in addition to the digestion and absorption of exogenous proteins into amino acids, the gluconeogenesis pathway in the liver can also convert pyruvate into amino acids. These amino acids are then transported via the bloodstream to the mammary gland, where they are utilized for milk protein synthesis (Zhou et al., 2022). Unlike during the lactation period, during the dry period, buffaloes allocate more energy towards self-repair and emotional regulation, storing additional triglycerides in preparation for the energy demands of the next lactation cycle (Avilés et al., 2019). This phenomenon reflects the metabolic regulation and adaptive responses of buffaloes during different physiological stages, ensuring a sustained energy supply and milk synthesis.

In summary, we conducted a relatively comprehensive analysis of the microbial–host interaction mechanisms in the rumens of buffaloes during both the lactation and dry periods. However, the physiological state of buffaloes is a dynamic process, and further investigations are needed to explore the influence of additional factors on the rumen microbiota and metabolic pathways in the future.

## Conclusion

This study analysed the changes in rumen microorganisms, functional characteristics, serum metabolites, and metabolic pathways in buffaloes. During the lactation period, the interplay between the rumen microbiota and host metabolism enhances the utilization of triglycerides and promotes the synthesis of amino acids, sugars, and other essential substances, thereby supporting milk production. During the dry period, microbial functions and metabolite profiles contribute to maintaining intestinal immune function and support lipid accumulation in the host. These findings provide a molecular basis for further elucidating the nutritional mechanisms driving host-mediated rumen microbiota remodeling to support buffalo lactation.

## Data Availability

The original contributions presented in the study are publicly available. This data can be found here: https://www.ncbi.nlm.nih.gov/, accession number: PRJNA656389.

## Footnotes

^1^ https://www.metaboanalyst.ca/

^2^ https://www.genome.jp/kegg/

^3^ https://metorigin.met-bioinformatics.cn/home/

## Glossary

5-HIAA: 5-Hydroxyindoleacetic acid
5-HTP: 5-Hydroxytryptophan
ALT: Glutamic pyruvic transaminase
AST: Glutamic oxaloacetic transaminase
CHOL: Cholesterol
DP: Dry period
Glu: Glucose
GLY: Glycolytic pathway
GNG: Gluconeogenesis pathway
HDL: High-density lipoprotein
I3M: Indole-3-methylacetate
IgA: Immunoglobulin A
LDA: Linear discriminant analysis
LDL: Low-density lipoprotein
LEfSe: Linear discriminant analysis effect size
LP: Lactation period
OPLS-DA: Orthogonal partial least squares discriminant analysis
TCA: Tricarboxylic acid cycle
TG: Triglycerides
Urea: Urea nitrogen
VIP: Variable importance in projection
