# Supplementary material for: Host-driven remodeling of rumen microbiota supports lactation metabolism in buffalo
Source: Front Microbiol. 2025 Jun 25;16:1617388. doi: 10.3389/fmicb.2025.1617388 (PMC12237897; doi:10.3389/fmicb.2025.1617388)

**Table S1**. Collection of rumen fluid samples during Lactation and Dry periods.

| Variety | Sample number | Sample classification | Period |
| --- | --- | --- | --- |
| Buffalo | S-1_BDME192032754-1a | Rumen fluid | Lactation period |
| Buffalo | S-2_BDME192032755-1a | Rumen fluid | Lactation period |
| Buffalo | S-3_BDME192032756-1a | Rumen fluid | Lactation period |
| Buffalo | S-4_BDME192032757-1a | Rumen fluid | Lactation period |
| Buffalo | S-5_BDME192032758-1a | Rumen fluid | Lactation period |
| Buffalo | S-6_BDME192032759-1a | Rumen fluid | Lactation period |
| Buffalo | S-7_BDME192032760-1a | Rumen fluid | Lactation period |
| Buffalo | S-8_BDME192032761-1a | Rumen fluid | Lactation period |
| Buffalo | S-9_BDME192032762-1a | Rumen fluid | Lactation period |
| Buffalo | S-11_BDME192032764-1a | Rumen fluid | Lactation period |
| Buffalo | S-12_BDME192032765-1a | Rumen fluid | Lactation period |
| Buffalo | S-13_BDME192032766-1a | Rumen fluid | Lactation period |
| Buffalo | S-14_BDME192032767-1a | Rumen fluid | Lactation period |
| Buffalo | S-15_BDME192032768-1a | Rumen fluid | Lactation period |
| Buffalo | S-16_BDME192032769-1a | Rumen fluid | Lactation period |
| Buffalo | S-17_BDME192032770-1a | Rumen fluid | Lactation period |
| Buffalo | S-18_BDME192032771-1a | Rumen fluid | Lactation period |
| Buffalo | S-19_BDME192032772-1a | Rumen fluid | Lactation period |
| Buffalo | S-20_BDME192032773-1a | Rumen fluid | Lactation period |
| Buffalo | S-21_BDME192032774-1a | Rumen fluid | Lactation period |
| Buffalo | S-22_BDME192032775-1a | Rumen fluid | Lactation period |
| Buffalo | S-23_BDME192032776-1a | Rumen fluid | Lactation period |
| Buffalo | S-24_BDME192032777-1a | Rumen fluid | Lactation period |
| Buffalo | S-25_BDME192032778-1a | Rumen fluid | Lactation period |
| Buffalo | S-26_BDME192032779-1a | Rumen fluid | Lactation period |
| Buffalo | S-27_BDME192032780-1a | Rumen fluid | Lactation period |
| Buffalo | S-28_BDME192032781-1a | Rumen fluid | Lactation period |
| Buffalo | S-63_BDME192032815-1a | Rumen fluid | Lactation period |
| Buffalo | S-64_BDME192032816-1a | Rumen fluid | Lactation period |
| Buffalo | S-29_BDME192032782-1a | Rumen fluid | Dry period |
| Buffalo | S-30_BDME192032783-1a | Rumen fluid | Dry period |
| Buffalo | S-31_BDME192032784-1a | Rumen fluid | Dry period |
| Buffalo | S-32_BDME192032785-1a | Rumen fluid | Dry period |
| Buffalo | S-33_BDME192032786-1a | Rumen fluid | Dry period |
| Buffalo | S-34_BDME192032787-1a | Rumen fluid | Dry period |
| Buffalo | S-36_BDME192032788-1a | Rumen fluid | Dry period |
| Buffalo | S-37_BDME192032789-1a | Rumen fluid | Dry period |
| Buffalo | S-38_BDME192032790-1a | Rumen fluid | Dry period |
| Buffalo | S-39_BDME192032791-1a | Rumen fluid | Dry period |
| Buffalo | S-40_BDME192032792-1a | Rumen fluid | Dry period |
| Buffalo | S-41_BDME192032793-1a | Rumen fluid | Dry period |
| Buffalo | S-42_BDME192032794-1a | Rumen fluid | Dry period |
| Buffalo | S-43_BDME192032795-1a | Rumen fluid | Dry period |
| Buffalo | S-44_BDME192032796-1a | Rumen fluid | Dry period |
| Buffalo | S-45_BDME192032797-1a | Rumen fluid | Dry period |
| Buffalo | S-46_BDME192032798-1a | Rumen fluid | Dry period |
| Buffalo | S-47_BDME192032799-1a | Rumen fluid | Dry period |
| Buffalo | S-48_BDME192032800-1a | Rumen fluid | Dry period |
| Buffalo | S-49_BDME192032801-1a | Rumen fluid | Dry period |
| Buffalo | S-50_BDME192032802-1a | Rumen fluid | Dry period |
| Buffalo | S-51_BDME192032803-1a | Rumen fluid | Dry period |
| Buffalo | S-52_BDME192032804-1a | Rumen fluid | Dry period |
| Buffalo | S-53_BDME192032805-1a | Rumen fluid | Dry period |
| Buffalo | S-54_BDME192032806-1a | Rumen fluid | Dry period |
| Buffalo | S-55_BDME192032807-1a | Rumen fluid | Dry period |
| Buffalo | S-56_BDME192032808-1a | Rumen fluid | Dry period |
| Buffalo | S-57_BDME192032809-1a | Rumen fluid | Dry period |
| Buffalo | S-58_BDME192032810-1a | Rumen fluid | Dry period |
| Buffalo | S-59_BDME192032811-1a | Rumen fluid | Dry period |
| Buffalo | S-60_BDME192032812-1a | Rumen fluid | Dry period |
| Buffalo | S-61_BDME192032813-1a | Rumen fluid | Dry period |
| Buffalo | S-62_BDME192032814-1a | Rumen fluid | Dry period |

**Table S2** Collection of serum samples samples during Lactation and Dry periods.

| Variety | Sample number | Sample classification | Period |
| --- | --- | --- | --- |
| Buffalo | L1 | Blood serum | Lactation period |
| Buffalo | L2 | Blood serum | Lactation period |
| Buffalo | L3 | Blood serum | Lactation period |
| Buffalo | L4 | Blood serum | Lactation period |
| Buffalo | L5 | Blood serum | Lactation period |
| Buffalo | L6 | Blood serum | Lactation period |
| Buffalo | L7 | Blood serum | Lactation period |
| Buffalo | L8 | Blood serum | Lactation period |
| Buffalo | L10 | Blood serum | Lactation period |
| Buffalo | d1 | Blood serum | Dry period |
| Buffalo | d2 | Blood serum | Dry period |
| Buffalo | d4 | Blood serum | Dry period |
| Buffalo | d5 | Blood serum | Dry period |
| Buffalo | d6 | Blood serum | Dry period |
| Buffalo | d7 | Blood serum | Dry period |
| Buffalo | d8 | Blood serum | Dry period |
| Buffalo | d9 | Blood serum | Dry period |
| Buffalo | d10 | Blood serum | Dry period |


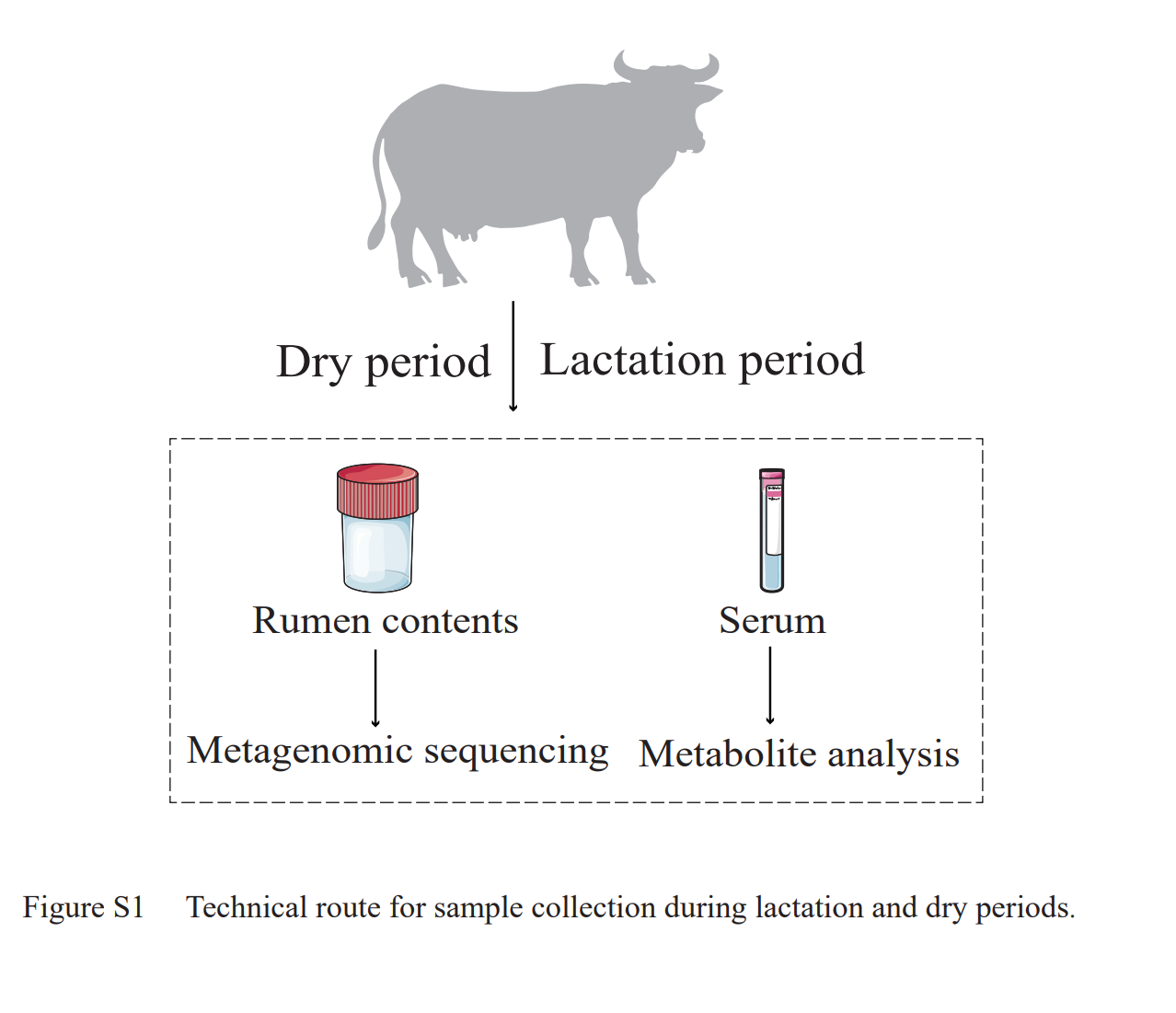


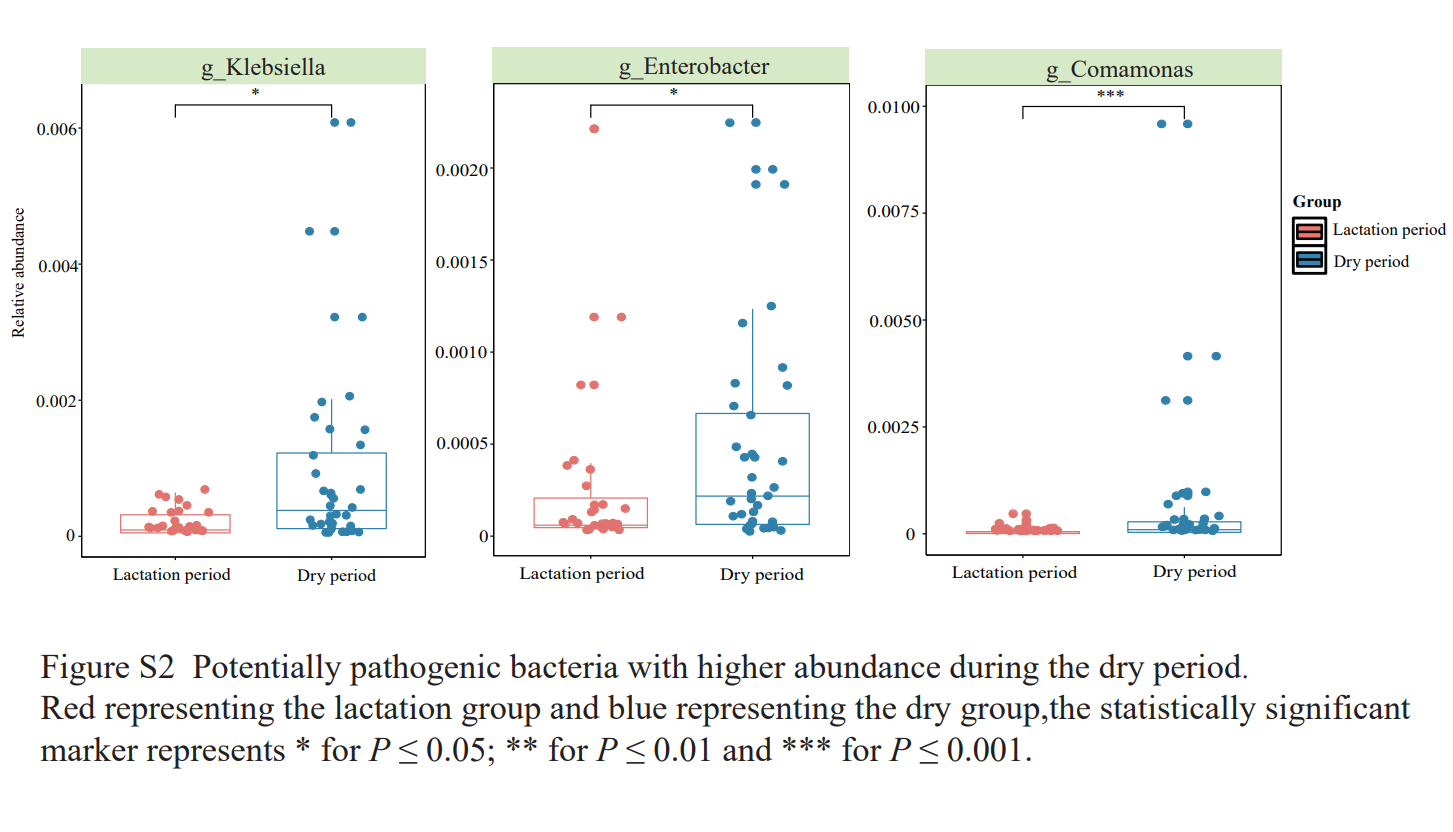


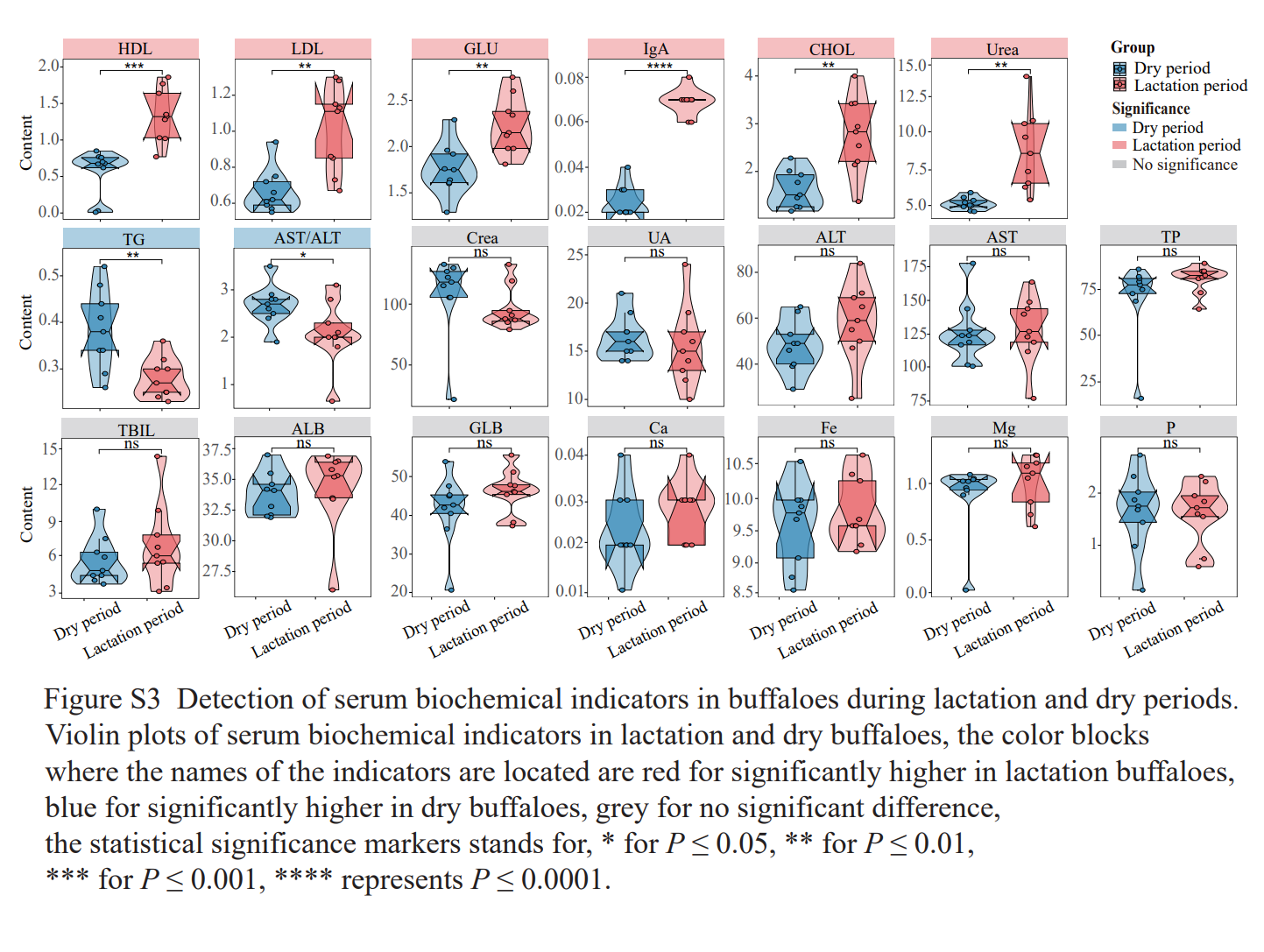


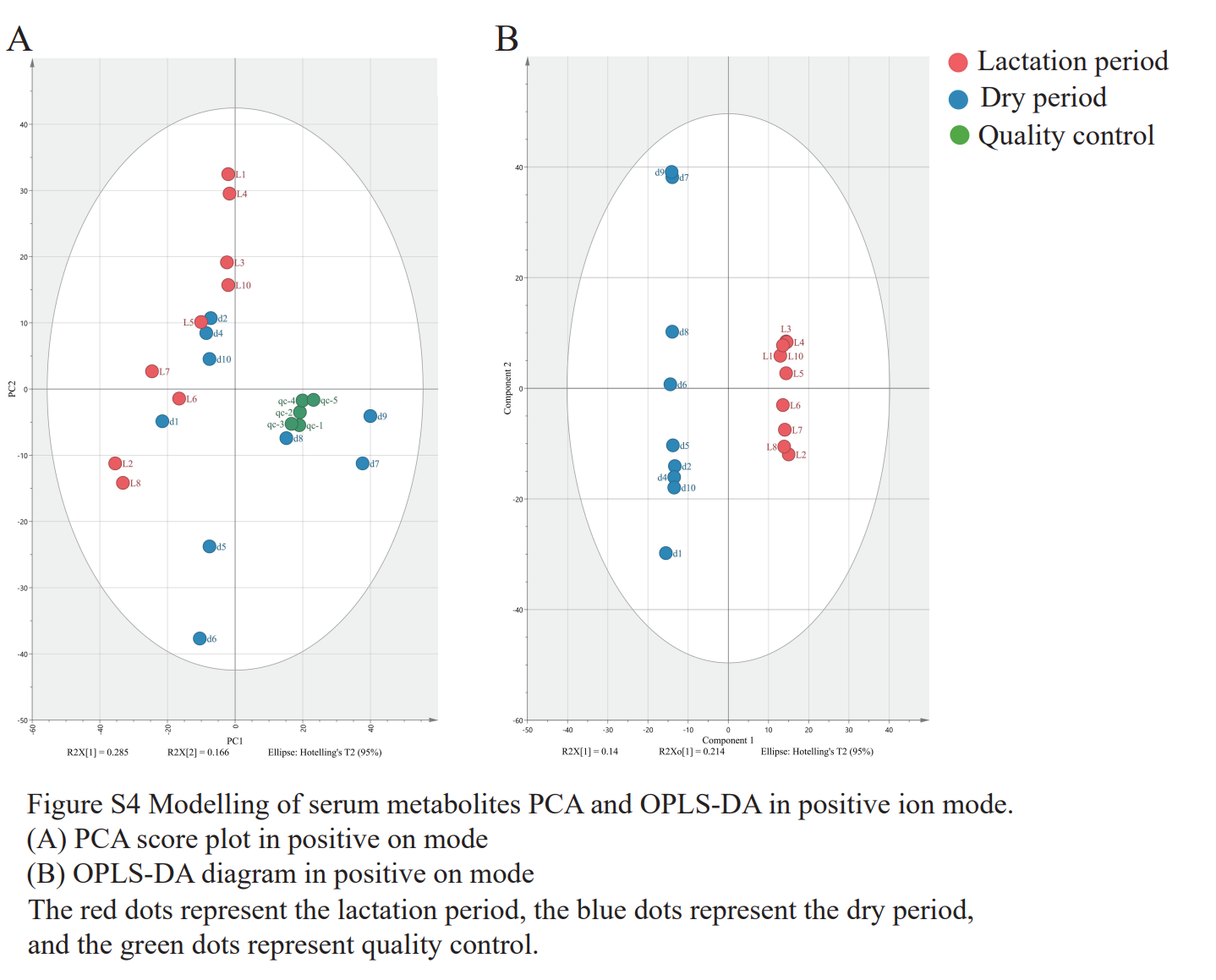

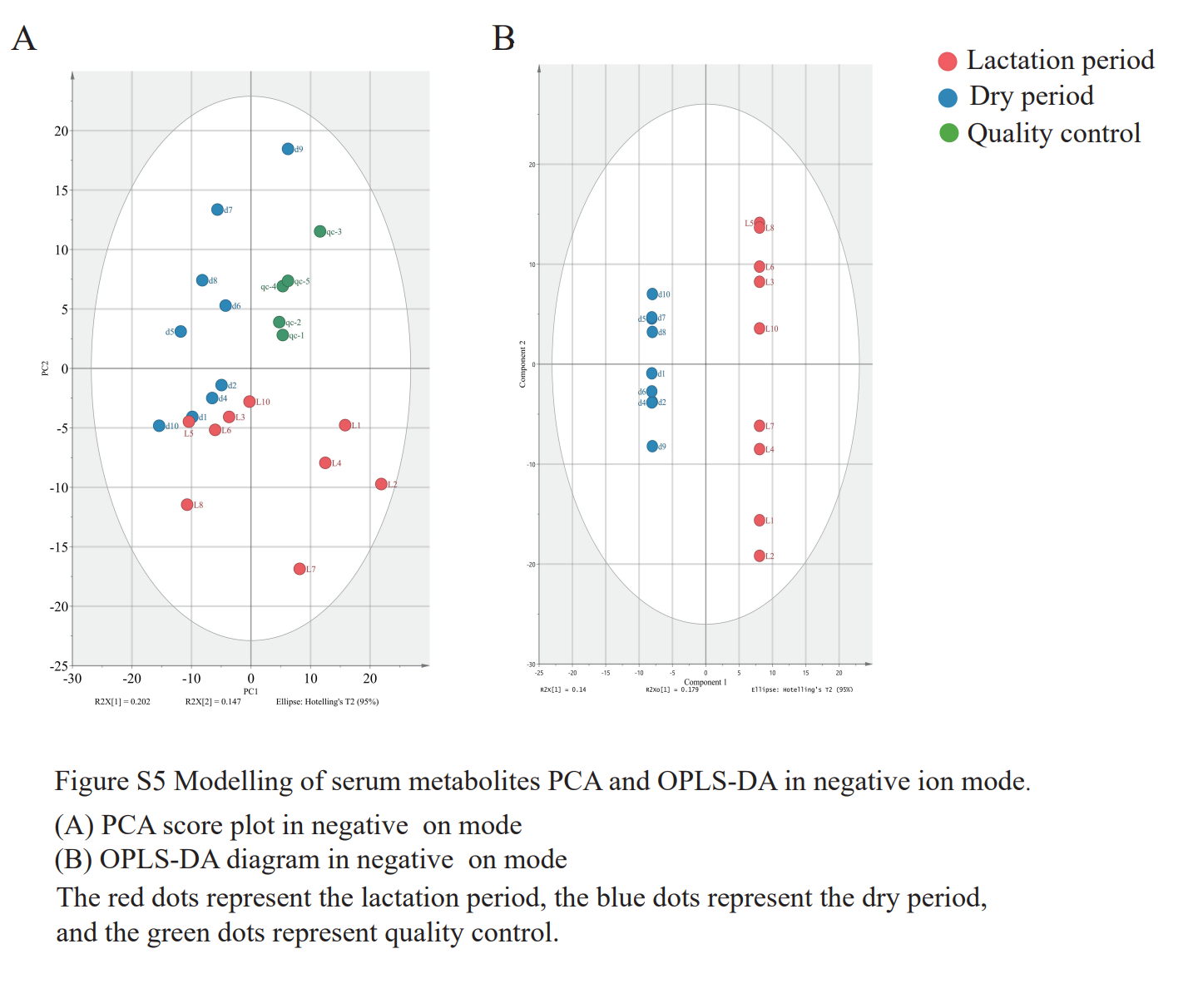

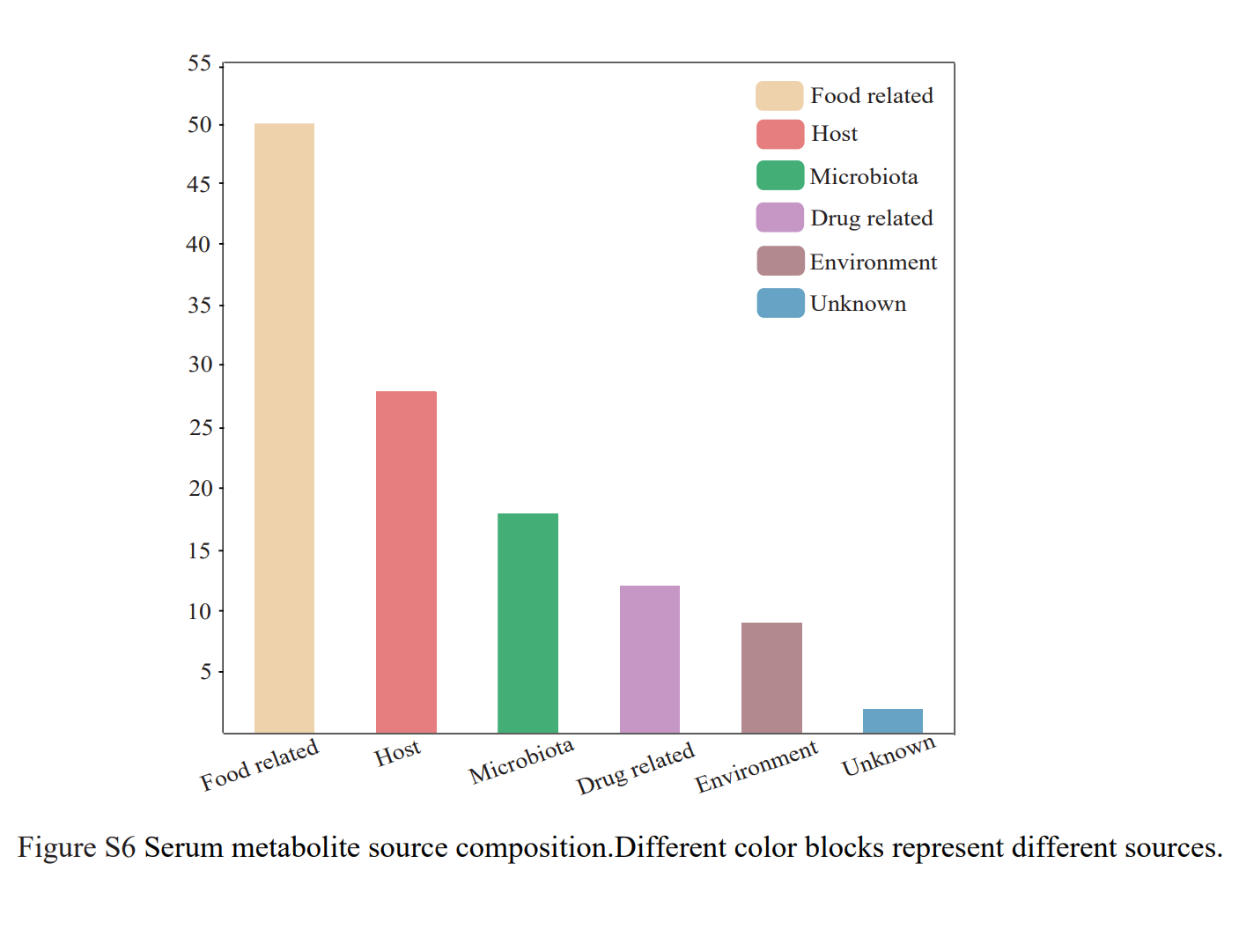

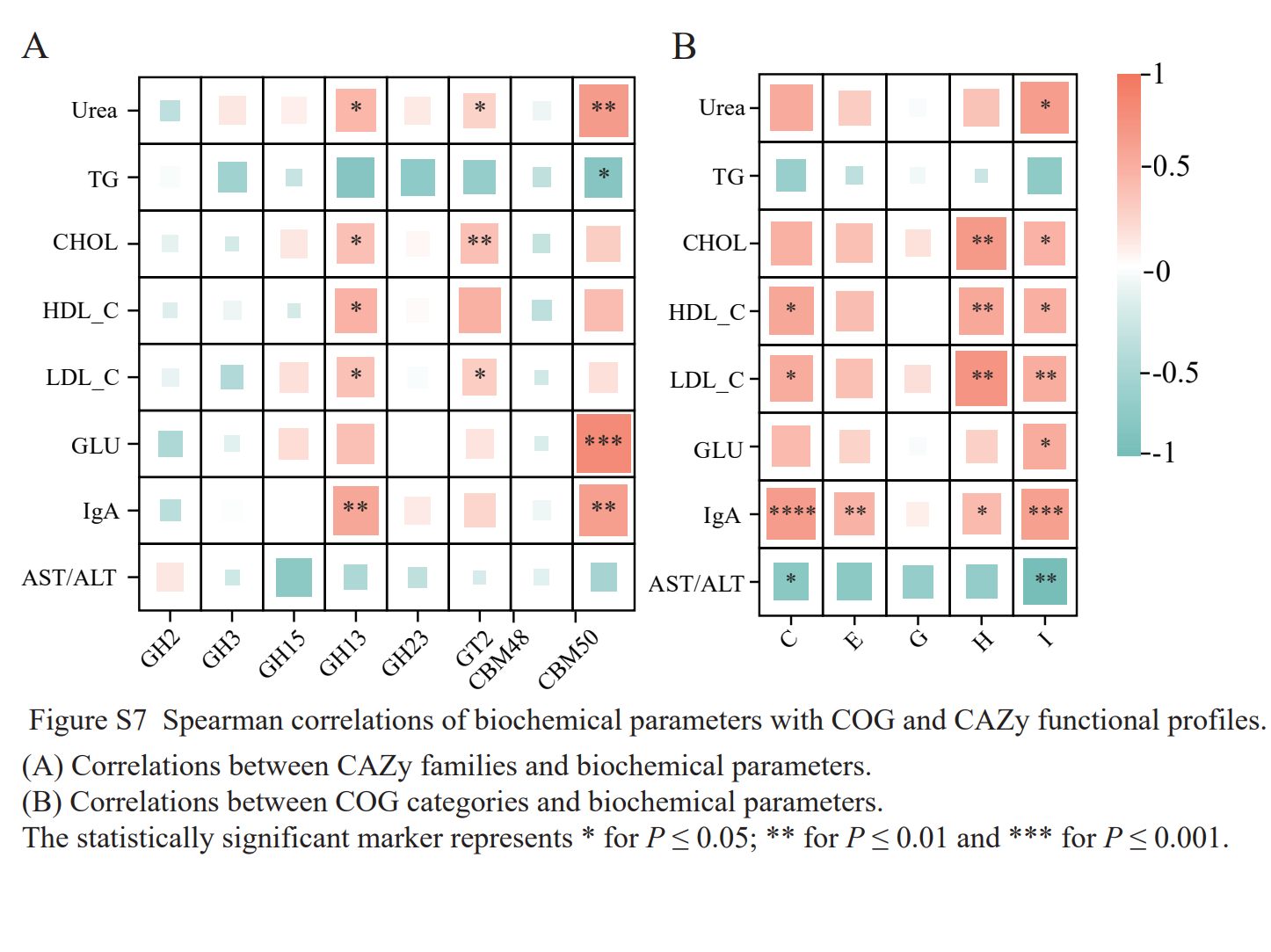

Supplement: Supplementary file 1 [file Supplementary_file_1.docx]
